# Combining Laser-Induced Graphene with Kirigami for Transparent Flexible Electromagnetic Interference Shielding

**DOI:** 10.1021/acsaenm.5c00861

**Published:** 2025-12-17

**Authors:** Mirza Sahaluddin, Mingxuan Li, Mehdi Zarei, Paul W. Leu, Mostafa Bedewy

**Affiliations:** † Department of Mechanical Engineering and Materials Science, 6614 University of Pittsburgh, 3700 O’Hara Street, Pittsburgh, Pennsylvania 15261, United States; ‡ Department of Chemical and Petroleum Engineering, University of Pittsburgh, Pittsburgh, Pennsylvania 15261, United States; § Department of Industrial Engineering, University of Pittsburgh, Pittsburgh, Pennsylvania 15261, United States

**Keywords:** graphene, laser processes, electromagnetic interference, flexible electronics, transparent conductors

## Abstract

This study presents a method for creating effective electromagnetic interference (EMI) shields that are transparent, lightweight, and flexible, which is vital to address the needs of emerging flexible electronics. By integrating laser-induced graphene (LIG) with kirigami—a technique of cutting and folding polymer films—spatial patterns of highly conductive 3D porous graphene is produced. The entire fabrication utilizes a single laser system for both LIG and cutting, which makes the direct-write process versatile and scalable. The resulting 3D graphene is highly conductive with resistance under 25 ohm/mm and good quality with G/D ratio at 1.66 and 2D/G ratio at 0.46. The films achieve an EMI shielding efficiency (SE) over 50 dB at a low density of 0.04 g/cm^∧^3. By leveraging the kirigami process to tune the SE and transparency, we achieve an SE of 17 dB while maintaining over 80% transparency, which exceeds previously reported values of 2D graphene. Additionally, our results address challenges in the flexibility and weight of EMI shields, achieving an exceptional EMI specific shielding efficiency (SSE) of 1362.2 dB cm^3^/g, competing with the previously reported values across thicknesses ranging from 10 to several hundred micrometers.

## Graphical abstract

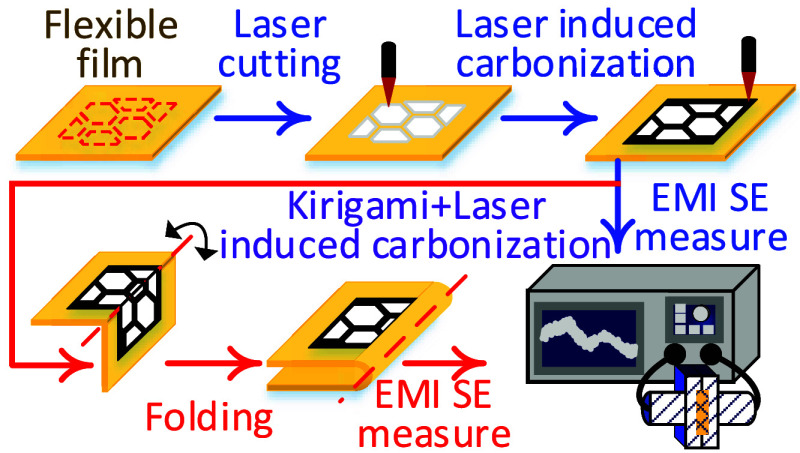

## Introduction

The rapid evolution of electronic gadgets and communication technology has resulted in a surge in the concentration of electromagnetic energy in the natural surroundings.^1,2^ This not only triggers electromagnetic interference (EMI), disturbing the operation of electronic devices, but also introduces a potential risk to human health.^3–5^ To mitigate the risks of electromagnetic radiation, an effective strategy is to employ materials designed for impeding the propagation of radiation which are capable of electromagnetic shielding. These materials arrest the spread of electromagnetic waves by reflecting and absorbing them.^6–8^

Importantly, there is a growing demand for lightweight EMI shielding materials with both high transparency and flexibility. These attributes are important in various applications such as aerospace equipment, display windows and wearable devices.^1,9–14^ Currently, many EMI shields rely on the use of metals, but their high density, rigidity and opaqueness greatly limit their adoption in emerging applications. Achieving both flexibility and transparency with metals necessitates thickness to be very low; for instance, silver films require thicknesses below 10 nm to reach desirable levels of transparency and flexibility.^15^ Consequently, metal-based EMI shields involve multiple manufacturing steps such as photolithography, reactive-ion etching and ink preparation,^16–19^ which complicates the fabrication. Furthermore, the use of highly conductive metals leads to undesired secondary electromagnetic radiation due to the reliance on electromagnetic radiation reflection.^20,21^ In addition, a panoply of different nanomaterials such as MXenes have also been proposed to achieve transparent EMI shielding. For instance, Yun et al. report a 1 dB EMI SE for a one layer MXene film with 90% transparency, whereas nine-layer MXene film warrants 10 dB at the cost of reduced transparency to 45%.^15^ While metal-based EMI shields and MXenes provide impressive shielding effectiveness, these solutions often sacrifice either transparency or ease/cost of manufacturing.

Carbon-based materials offer an alternative for low-density, lightweight EMI shielding, which is relevant for flexible electronics and aerospace applications. Carbon nanotubes (CNTs), carbon nanofibers (CNFs), graphene nanosheets, and carbon black are all alternative highly conducive materials..^22–25^ While graphene, in particular, is highly promising to supplant metals in pursuit of lightweight and high-performance EMI shielding,^26,27^ the flat and thin nature of graphene sheets grown by chemical vapor deposition on rigid substrates limits its integration with polymeric materials in flexible devices.^28,29^ On the other hand, multistep processing methods of three-dimensional conductive structures, such as spatially distributed CNTs, allow the integration in polymers, such as additives in polymeric composites. The high aspect ratio and 3D distribution allows for attaining significant electrical conductivity with a limited amount of CNTs.^30–32^ Zhao et al. integrated CNTs into a sponge-like polymer and assessed their capability for EMI shielding, revealing an approximate 18 dB SE within the 8–12 GHz frequency range.^33^ Similarly, Nam et al. produced epoxy-based polymeric composites incorporating 5 wt % CNTs, yielding an EMI SE of about 12 dB for frequencies spanning 2–5 GHz.^34^ Efforts to fabricate 3D networked graphene structures commonly rely on support materials like Ni foam,^35^ porous Cu foil,^36^ and hydrogels,^37^ which provide a structural template or reinforcement during the graphene formation process. These fabrication steps entail intricate and costly preparation processes that challenge mass production due to the involvement of multiple steps,^38^ extended durations,^39^ elevated temperatures^40^ and/or ultrapure atmospheres.^41^ Hence, there is an increasing need to find materials that combine high EMI shielding performance with lightweight, flexibility, and stability on flexible and wearable devices.

Laser-induced graphene (LIG) offers a streamlined, scalable, and economically viable manufacturing process for directly patterning 3D porous graphene on polymer films,^42^ without the need for transfer steps. This one-step process has shown promising results in diverse applications, such as sensors, supercapacitors, and antibacterial membranes.^43–49^ Xu et al.^50^ investigated the potential of LIG for EMI shielding, demonstrating an EMI SE of over 40 dB for a pristine LIG. To further improve the SE, composites are made of LIG with materials such as MXenes and iron oxide nanoparticles.^51–54^ While these findings highlight LIG’s promise in EMI shielding applications, the resulting shields lack transparency. Thus, making LIG patterns both functional and transparent is still needed for applications requiring optical transparency, such as screens, windows and see-through shields.

In this study, we introduce a method for fabricating transparent and flexible EMI shields through a scalable, efficient and versatile process that merges LIG with kirigami, a technique involving cutting and folding of films. Kirigami techniques, widely applied in flexible electronics for frequency selective surfaces,^55^ strain sensors^56^ and supercapacitors,^57^ are here uniquely combined with LIG to pioneer a rapid manufacturing of transparent EMI shields directly on polyimide films. Utilizing a single laser for both LIG and cutting in this direct-write process, our method not only simplifies fabrication but also enables adaptability to a variety of substrates with customizable geometries. This process results in an exceptional EMI SE, surpassing 50 dB at a remarkably low density of 0.04 g/cm^3^. The real novelty comes with the incorporation of kirgami, which allows us to tune the EMI SE and transparency. Our developed shields can achieve an SE of over 17 dB while maintaining over 80% transparency.

## Experimental Section

This section describes the fabrication of unfolded and folded EMI shields, followed by their structural, electrical, and optical characterization, as well as the procedures used to quantify transparency, geometric deviations, and EMI shielding effectiveness.

### Fabrication of EMI Shields

The EMI shields were fabricated from commercial polyimide (PI) films (CS Hyde; 250 μm thick, item no. 18–10F–P, for unfolded samples and 200 μm thick, item no. 18–8F–P, for folded samples). Prior to laser processing, the PI sheets were rinsed sequentially with acetone and isopropyl alcohol, then dried in air to ensure a clean surface. All laser processing was performed in ambient air using a CO_2_ laser cutter/engraver (Full Spectrum Laser Pro-Series 20 × 12, wavelength 10.6 μm, maximum power 45 W) equipped with a 1.5 in. focal length lens and a motorized XY stage (maximum speed 500 mm s^–1^ in the horizontal X direction). As illustrated in Figure 1, the fabrication comprised two main steps. In the first step, the honeycomb mesh geometry consisting of hexagonal cuts was defined in vector mode. The cuts were patterned using a laser power of 23.2 W, a speed of 15.8 mm s^–1^, and 3 passes, with the beam in focus. The hexagonal honeycomb pitch and width as shown in Figure S1 were varied according to eq S1 to achieve different optical transparencies. The corresponding geometric parameters for each design are summarized in Table S1. For folded samples, an additional laser pass was used to introduce a partial-thickness crease along the symmetry axis between the mirrored halves of the pattern (Figure 1), which served as the folding hinge. In the subsequent step, the PI regions were converted to LIG using raster mode. Unless otherwise stated, the laser power, head speed, defocus, and raster line spacing were set to 12.5 W, 111 mm s^–1^, a defocus of 6 mm above the focal plane, and 355 μm, respectively, with 2 passes. These conditions, identified based on our previous optimization studies,^58–61^ consistently yield a continuous, conductive LIG layer on the PI. The same LIG-writing parameters were used for both unfolded and folded samples.

**Figure 1:**
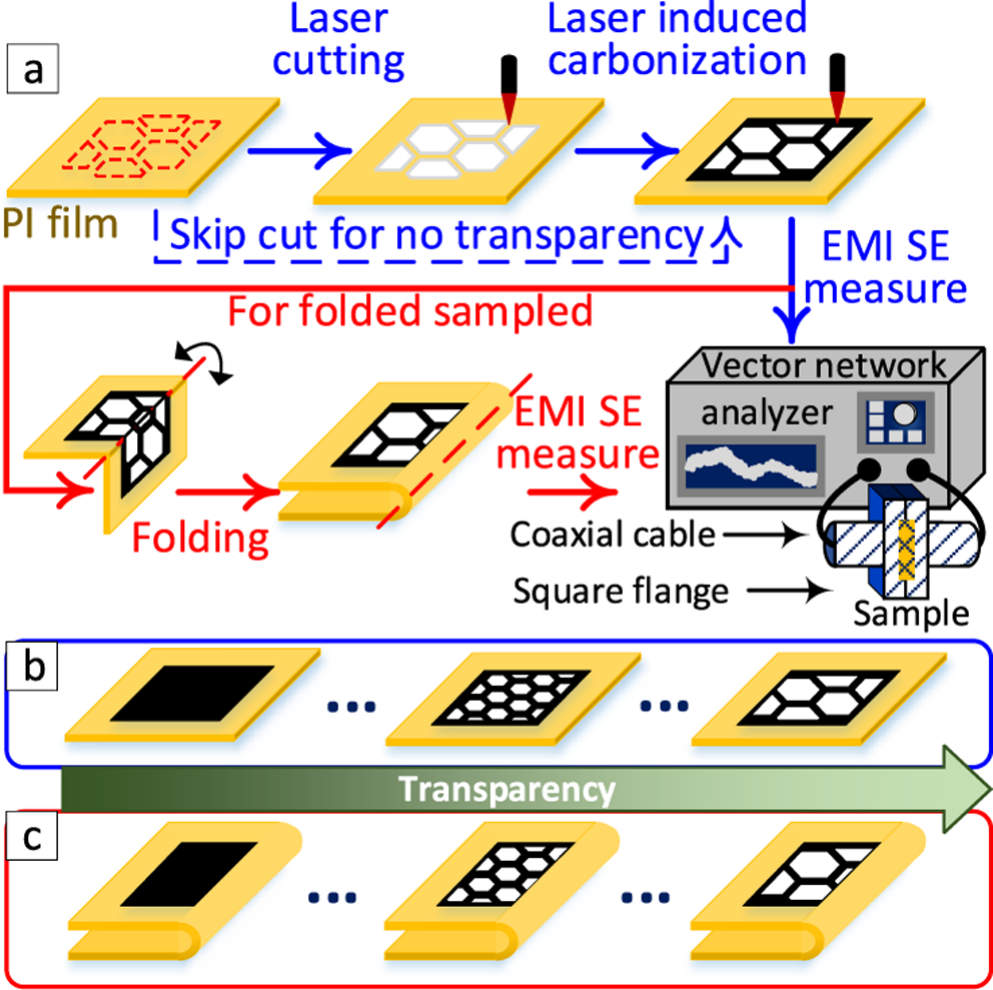
(a) Steps involved during the fabrication process of LIG EMI shields by kirigami for both unfolded (blue steps) and folded (red steps) samples, (b, c) schematic illustrating the varying degrees of transparency achieved by designing different cut patterns and areas for unfolded (b) and folded (c) samples.

### Material Characterization

The structural and morphological characterization of the transparent and flexible films was carried out using scanning electron microscopy (SEM) and Raman spectroscopy. SEM images were obtained using Zeiss Sigma VP, which allowed for the visualization of the surface and cross-sectional morphology to measure the thickness. No additional coating of conductive layer was required on the films to prevent surface charging as the films are highly conductive. The thickness of the porous LIG region above the polyimide substrate was measured using ImageJ, by drawing line profiles normal to the interface at more than 15 different regions per sample. The mean thickness was calculated from these measurements. A representative cross-sectional SEM image used for this analysis is shown in Figure S6­(e). Zeiss smartzoom 5 was used for obtaining the optical microscopy images. The acquisition of Raman spectra was conducted utilizing the XplorA Raman-AFM/TERS microscope system. The experimental setup involved the utilization of a laser with a wavelength of 473 nm operating at a power level of 25 mW. Areal Raman mapping measurements were conducted by Horiba LabRAM Soleil Raman Microscope using a 532 nm excitation source to evaluate the uniformity of the LIG structure near and away the kirigami cuts. The measurements were performed under ambient conditions at room temperature.

### Electrical Measurements

To assess the electrical conductivity of the fabricated materials, Keithley two-point probe meter (model: 2100) with a detection limit of 100 MΩ was utilized. Furthermore, a four-point probe measurement (Keysight B1500A) was conducted to determine the sheet resistances through the van der Pauw method. To ensure improved contact between the probe terminals and the LIG at the measurement spots, a silver paste (PELCO Conductive silver Paint) was applied.

### Quantifying Geometric Deviations

Sample warping was characterized by utilizing the ARTEC SPACE SPIDER 3D SCANNER to obtain the topographical data of both EMI samples and bare PI substrates. The assessment of warping levels is analyzed by measuring the maximum profile height, calculated as the difference between the magnitude of the maximum and minimum of surface heights. To obtain the complete set of 3D coordinates of each sample, the STL file obtained from the 3D scanner is imported into MATLAB. Subsequent steps encompassed tilt and rotation adjustments with reference to the average surface height for calculating the maximum profile height.

### Transparency Analysis

The transparency analysis started by using MATLAB software for image processing of kirigami patterned LIG samples. Digital images of the samples were captured using a high-resolution camera (12 megapixels iPhone 11), and these images were processed using MATLAB algorithms (image processing toolbox) to quantitatively evaluate the transparency of the EMI shields based on area fractions of LIG and cut areas. Each image is read and converted to grayscale using *rgb2gray*, followed by binarization with *imbinarize* to isolate the holes. The *bwlabel* function labels individual holes, and *regionprops* calculates their areas. The total area of the holes is summed, and the total pixel count of the image is determined to calculate transparency as the ratio of the total hole area to the total image area. More details are provided in Figure S2 and Table S1. The transmittance of fully covered LIG areas was measured using a UV–Vis spectrophotometer (PerkinElmer Lambda 750) equipped with a 100 mm integrating sphere over the 400–800 nm wavelength range.

### EMI SE Measurement

The EMI SE was measured using a rectangular waveguide transmission line method, in which the vector network analyzer (HP 8722D) was connected to the waveguide test fixture through coaxial-to-waveguide adapters, as shown in Figure 1. Pasternack waveguide fixtures were used for the X-band (8–12 GHz, WR90, 22.86 × 10.16 mm^2^ aperture, model PEWCA1025) and Ku-band (12–18 GHz, WR62, 15.8 × 7.9 mm^2^ aperture, model PEWCA1023). The samples were larger than the waveguide aperture and were positioned between the two flange faces, which were then secured firmly with screws and nuts to ensure tight clamping and minimize any electromagnetic leakage. The scattering parameters *S*_11_ and *S*_21_ were recorded and the transmission (*T*), reflection (*R*) and absorption (*A*) coefficients were calculated using the following relationships

1 $$T={\left|S_{21}\right|}^{2}$$

2 $$R={\left|S_{11}\right|}^{2}$$

3 T+R+A=1

The EMI SE and its components of reflection (SE_R_) and absorption (SE_A_) are calculated using the relationships

4 $$\mathrm{SE}=-10\log_{10}T$$

5 $${\mathrm{SE}}_{R}=-10\log_{10}\left(1-R\right)$$

6 $${\mathrm{SE}}_{A}=\mathrm{SE}-{\mathrm{SE}}_{R}$$

For both the folded and unfolded samples, the density (ρ) used for calculating specific shielding efficiency (SSE) refers to the density of the porous LIG layer only, excluding the PI substrate.

7 SSE=SE/ρ

## Results and Discussion

The manufacturing process is illustrated in Figure 1a. It details the (a) step-by-step procedure for creating transparent EMI shielding graphene in both their unfolded (Figure 1b) and folded (Figure 1c) forms for transparent EMI shielding measurements. The manufacturing process begins with a CO_2_ laser that irradiates a 10.6-μm beam onto a polyimide substrate. This is executed in vector mode to create hexagonally shaped cuts in a honeycomb pattern. Specific geometric designs as defined by the width of the graphene regions and the pitch of the hexagons are used to control the transparency levels of the resulting samples. These geometric parameters are illustrated in Figure S1 and the nominal transparency is calculated using eq S1. The hexagonal cuts are performed with controlled dimensions, allowing the fabrication of samples with varying nominal transparencies (Table S1).

After the vector-mode cutting (at low speed of 15.8 mm/s, power 23.2 W, at focus and three passes), the same laser machine switches to raster mode (at faster speed of 111 mm/s, power 12.5W and defocus 6 mm and two passes) to produce high-quality LIG on the polyimide substrate. The created transparent samples then undergo testing for their EMI shielding performance. For samples designed to have zero percent transparency, the vector mode cutting step is skipped. These samples proceed directly to performance assessment following the raster-mode operation, i.e., full coverage of LIG.

In order to fabricate samples with multiple LIG layers, we also utilize folding, as shown in Figure 1c. Samples are creased and folded along one side, generating a mirror image that includes both the LIG and the hexagonal cuts. The CO_2_ laser machine creates a crease across the plane of folding, positioned in line with the symmetry between the mirrored halves. This crease aids in folding the sample while preserving its transparency levels. The EMI shielding performance of these folded samples is also tested to assess their efficiency. Overall, our manufacturing approach is rapid and customizable. It employs a single laser system for both the LIG and cutting steps, resulting in transparent LIG-based EMI shields with any arbitrary geometry.

Laser parameters, such power and degree of beam defocus significantly affect the conductivity of LIG lines and areas.^58,61^ Here, we focus on three key laser-processing parameters (beam defocus, laser power, and the gap which is equivalent to the grating spacing between adjacent scan lines) and optimize them to achieve the lowest electrical resistance while minimizing geometric warping of the samples. Accordingly, as shown in Figure 2, we first optimize the process for LIG lines to achieve the lowest resistance per unit length. Second, we create areas using the raster mode with different line-to-line distances, referred to here as gaps, in order to create LIG areas with the lowest sheet resistance and minimal sample warping. Figure 2a shows that increasing beam defocus and power results in fabricating wider LIG lines (100s μm width) with lower resistance per unit length of the lines. Optical microscopy images of the lines at different levels of defocus are illustrated in Figure S9. As shown in our previous work, increasing beam defocus results in creating larger line widths and is critical for creating the desirable cellular network morphology, which is also the most electrically conductive.^58^ Combination of these properties is particularly important for fabricating the low-density LIG-based EMI shields with high SE in this work.

**Figure 2:**
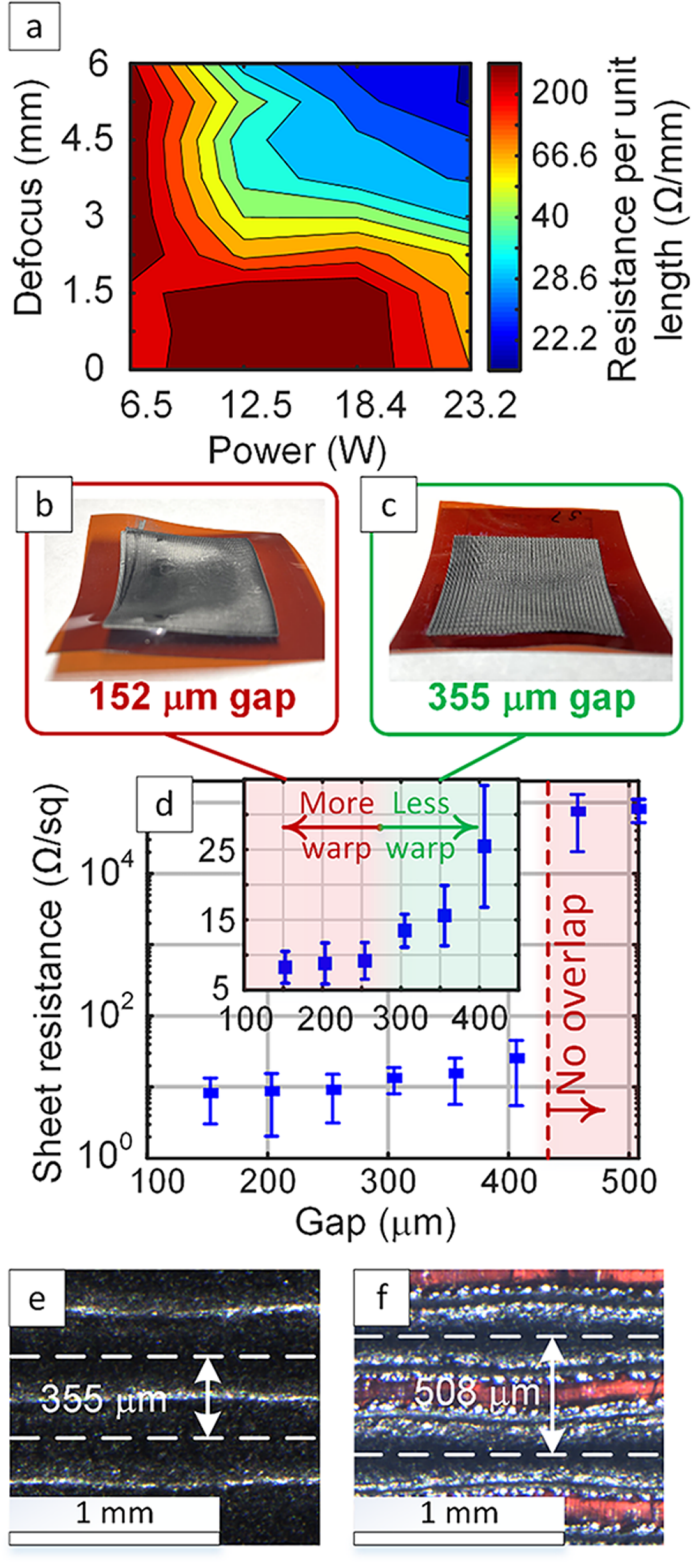
LIG process optimization for maximizing electrical conductivity. (a) a map showing the effect of changing beam defocus and power on the resistance-per-length measured for LIG lines obtained by CO2 laser irradiation at 111 mm/s. (b, c) Images showing the difference in sample warping for two samples lased using 152 and 355 μm line-to-line distance (gaps) in laser raster mode for creating LIG areas. (d) Gap-dependent change in sheet resistance of LIG areas fabricated using speed 111 mm/s, power 12.5 W, defocus 6 mm and two passes, showing the threshold for line overlap that is necessary to achieve high electrical conductivity. Inset: same data in (d) plotted using linear scale for the vertical axis instead of a logarithmic scale. (e, f) Optical microscopy images for LIG areas fabricated using the same laser conditions in (d) with gaps of 355 and 508 μm respectively, showing no line overlap in the case of 508 μm gap. The error bars represent the standard error based on three independent samples (*n* = 3).

To create uniform LIG areas the line-to-line distance of laser passes in the raster mode are also important for both minimizing the sheet resistance and reducing any geometric deviations of the sample after lasing (due to warping). Images in Figure 2b,c demonstrate the effect of gap on warping, showing that a smaller gap of 152 μm (Figure 2b) results in significant warping, while a larger gap of 355 μm (Figure 2c) results in much less warping. These deviations are quantified by using a 3D scanner to measure the 3D geometry after lasing, as shown in Figures S3–S5. This topographical data is used to analyze LIG samples fabricated with gaps ranging from 152 to 406.4 μm. Topography maps with a top view (Figure S3) and isometric view (Figure S4) are analyzed. In addition, a plot of maximum profile heights extracted from these measurements as a function of gap is included in Figure S5, as compared to bare PI. Generally, less warping manifests as a reduction in the maximum profile height with increasing gap. This correlation is caused by decrease in supplied laser fluence for larger gap distances compared to their smaller counterparts. Taken together, our results show that samples with gaps in the range of ≈ 300–400 μm exhibit the best balance of low sheet resistance and minimal warping, as shown in Figure 2d.

Beyond geometric distortion, the raster gap also influences the adhesion of LIG on the PI substrate. The adhesion between the LIG and PI is inherently strong owing to the *in situ* nature of LIG formation, where laser-induced carbonization of the polymer surface creates a continuous interfacial transition between the graphitized layer and the underlying PI. This seamless interface promotes strong adhesion and mitigates delamination from the substrate. However, at very small raster gaps (e.g., 0.152 mm), the cumulative laser fluence becomes sufficiently high to induce pronounced warping and partial delamination in localized regions (Figure S13). To reduce such effects while maintaining low sheet resistance, the laser power, scanning speed, defocus, and raster gap were optimized following our recent work.^58,60,62^ For reliable electrical and mechanical characterization, the lowest-gap sample and other highly warped specimens outside the optimal 300–400 μm range as discussed above were excluded from subsequent EMI shielding measurements to avoid artifacts arising from mechanical instability. Thus, the raster gap plays a dual role in governing both structural deformation and electrical performance.

Sheet resistance measurements for LIG areas fabricated using different gaps (Figure 2d) also show that there is a threshold value of gap (≈450 μm), above which there is no line-to-line overlap in LIG lines. Comparing optical microscopy images of two samples fabricated with different gaps show that a gap of 355 μm results in continuous areas with clear line overlap, while a gap of 508 μm results in no overlap between successive laser passes during rastering (Figure 2e,f). This lack of overlap explains the five-order-of-magnitude increase in sheet resistance observed in Figure 2d for gaps larger than ≈450 μm. SEM images in Figure S6 also shows the uniformity of the porous LIG morphology for the optimized gap of 355 μm. These results demonstrate that tuning beam defocus and laser power (Figures 2a and S9), together with the raster gap/grating spacing (Figures 2b–f, S3–S5 and S13), enables optimization of LIG line resistance, sheet resistance, and geometric stability for EMI shielding applications.

In addition to the electrical conductivity measurements, the geometry, nanoscale morphology and atomic structure of the LIG samples are thoroughly characterized, as shown in Figure 3. Several characterization techniques were combined, including optical microscopy, Raman spectroscopy, and scanning electron microscopy (SEM). Optical microscopy images are shown in Figure 3a, which provides insights into the pattern quality and key aspects of the obtained smooth edges and uniform web thicknesses. Furthermore, Figure 3b demonstrates the sample’s flexibility, revealing its suitability for applications requiring deformation or integration into flexible electronic devices. To evaluate the mechanical durability of the LIG films, a fully covered sample (3 cm × 3 cm) was subjected to cyclic bending up to 2000 cycles at a bending diameter of 8 mm, using sheet resistance as the assessment metric. The normalized resistance (*R*/*R*_0_) increased by less than 11% (Figure S12), indicating that the conductive network remained electrically stable under repeated deformation. As illustrated in the insets of Figure S12, optical microscopy images taken before and after the bending test revealed no visible cracks, delamination, or changes in surface morphology, confirming that the porous structure remained intact throughout cycling. Since optical transparency in patterned films is determined by the size and distribution of laser-cut openings, which are absent in the fully covered sample tested here, the transparency is expected to remain unchanged after bending. These results demonstrate the excellent mechanical robustness of the LIG film, consistent with previous reports on LIG exhibiting negligible resistance drift and preserved morphology after extensive bending cycles.^63,64^ Following optical microscopy, SEM imaging was carried out to elucidate the LIG morphologies close to the cut edge (Figure 3c­(iii)) and in the middle of the webs (Figure 3c­(iv)), as shown in Figure 3c. While subtle differences in surface porosity are noted, results generally show similar morphologies of 3D porous graphene in both cases.

**Figure 3:**
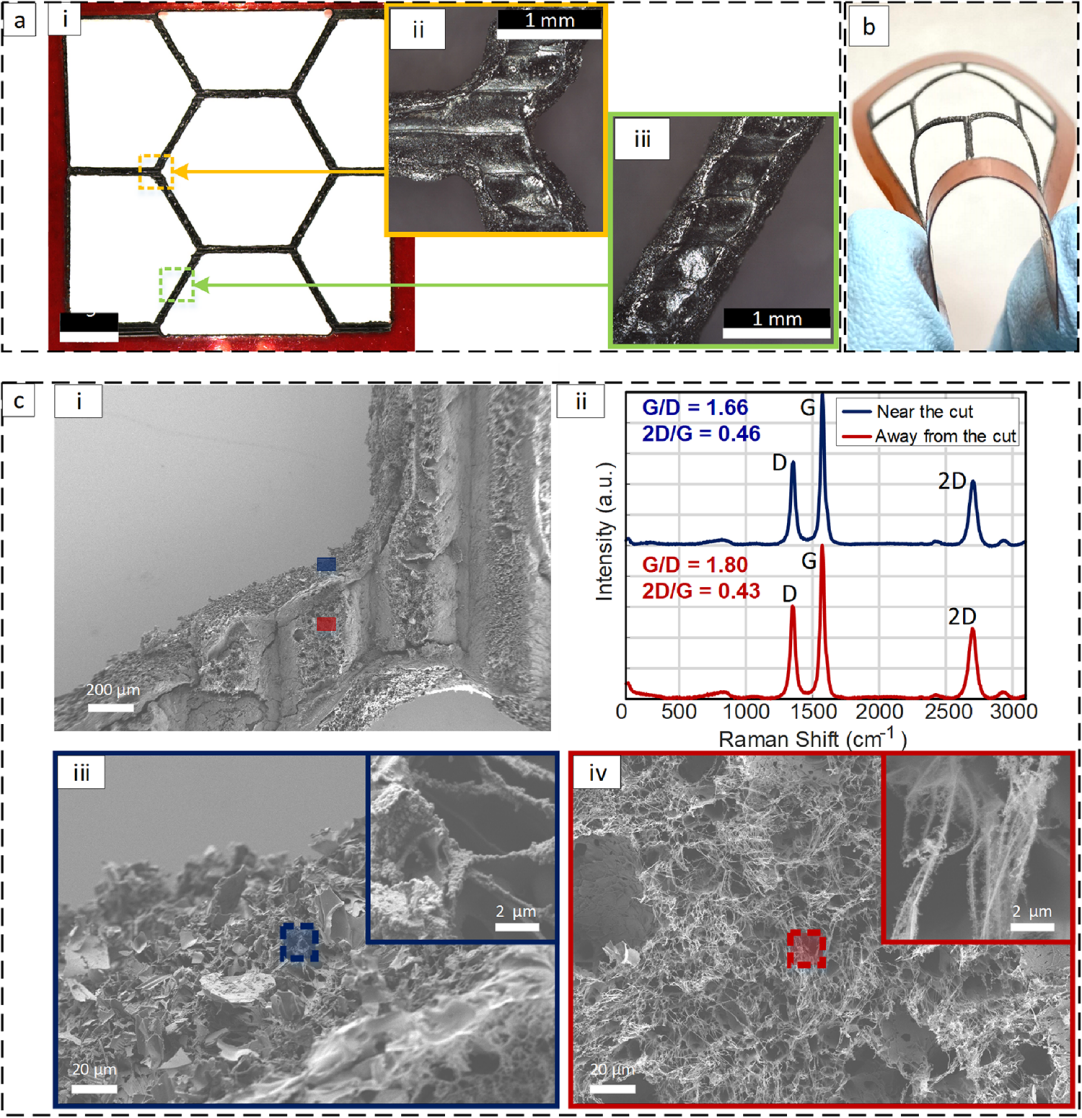
(a) Optical microscopy images of a representative sample (fabricated with laser parameters of speed 111 mm/s, power 12.5 W and defocus 6 mm and two passes; 88% transparency unfolded) with higher magnification images in a­(i) and a­(ii). (b) Illustration of sample flexibility. (c) Characterization near the cut and away from the cut. c­(i) low magnification SEM, showing the web and identifying the two point near the cut and away from the cut. c­(ii) Raman spectroscopy of the LIG near and away from the cut and c­(iii), c­(iv) Scanning electron microscopy image depicting the morphologies with enlarged view near and away from the cut, respectively.

Moreover, Raman spectroscopy was utilized to analyze the molecular structure of LIG. The spectra exhibit three characteristic peaks at approximately 1350, 1580 and 2700 cm^–1^, corresponding to the D, G, and 2D bands, respectively. The D band originates from defect-induced vibrations in sp^2^ carbon rings, while the G band represents the in-plane stretching of sp^2^ carbon–carbon bonds in graphitic domains.^65^ The ratio of their intensities (D/G or inversely G/D) provides an indication of the defect density and degree of graphitization, with higher G/D values suggesting improved crystalline order. The 2D band reflects the stacking order and layer number of graphene, where a higher 2D/G ratio and a smaller full width at half-maximum (fwhm) correspond to more ordered, few-layer graphene. When comparing the Raman features near the cut and away from the cut, both regions exhibit nearly identical characteristics. The G/D ratios were 1.66 and 1.80, while the 2D/G ratios were 0.46 and 0.43, respectively. The D, G, and 2D peak positions near and away from the cut were 1354 and 1349 cm^–1^, 1576 and 1573 cm^–1^, and 2703 and 2698 cm^–1^, respectively (Figure 3c­(ii)). Both areas also showed comparable peak sharpness, with FWHM­(G) values of 38.3 cm^–1^ and FWHM­(2D) values of 65.1 and 67.7 cm^–1^. These narrow and well-defined peaks, combined with consistent intensity ratios, indicate that the crystallinity, defect density, and number of graphene layers remain uniform near and away from the kirigami cuts. To further assess spatial uniformity, Raman mapping was performed over a 300 μm by 220 μm area, as shown in Figure S10. The 2D/G ratios were consistent near and away from the edges, confirming that the laser cutting process does not introduce measurable structural disorder in the LIG network.

Taken together, our results demonstrate remarkable uniformity of material morphology and atomic structure with no significant difference near and away the laser-cut edges. More SEM images are included in Figure S6, showing the uniformity of LIG morphology over large areas (without the effect of cutting), as well cross-sectional images showing the uniform thickness and confirming the LIG surface adhesion. Additionally, Figure S6c,d show SEM images of the small transition region between the area close to the laser-cut edges and the area farther away, revealing no significant difference in surface morphology. Hence, our LIG-based samples are promising for highly efficient and flexible EMI shielding in various practical applications.

EMI SE measurements for both our unfolded and folded transparent EMI shields are shown in Figure 4. EMI SE spectra over a frequency range of 8–18 GHz are shown in Figure 4a for the unfolded samples, and in Figure 4b for the folded samples. Here, we fabricate different samples having a variation of transparency ranging from 0% (LIG-covered polyimide film without any cutting), and 100% (bare polyimide film with a laser-cut square). It is worth noting here that the value of transparency of our various transparent EMI shields were calculated using MATLAB from optical image processing, as described in Figure S2 and Table S1, and were compared to the nominal transparency values calculated from the designed honeycomb geometry. Minor differences are observed between the predicted transparencies (based on designed geometry) and the transparency values experimentally obtained from image processing of the samples after manufacturing.

**Figure 4:**
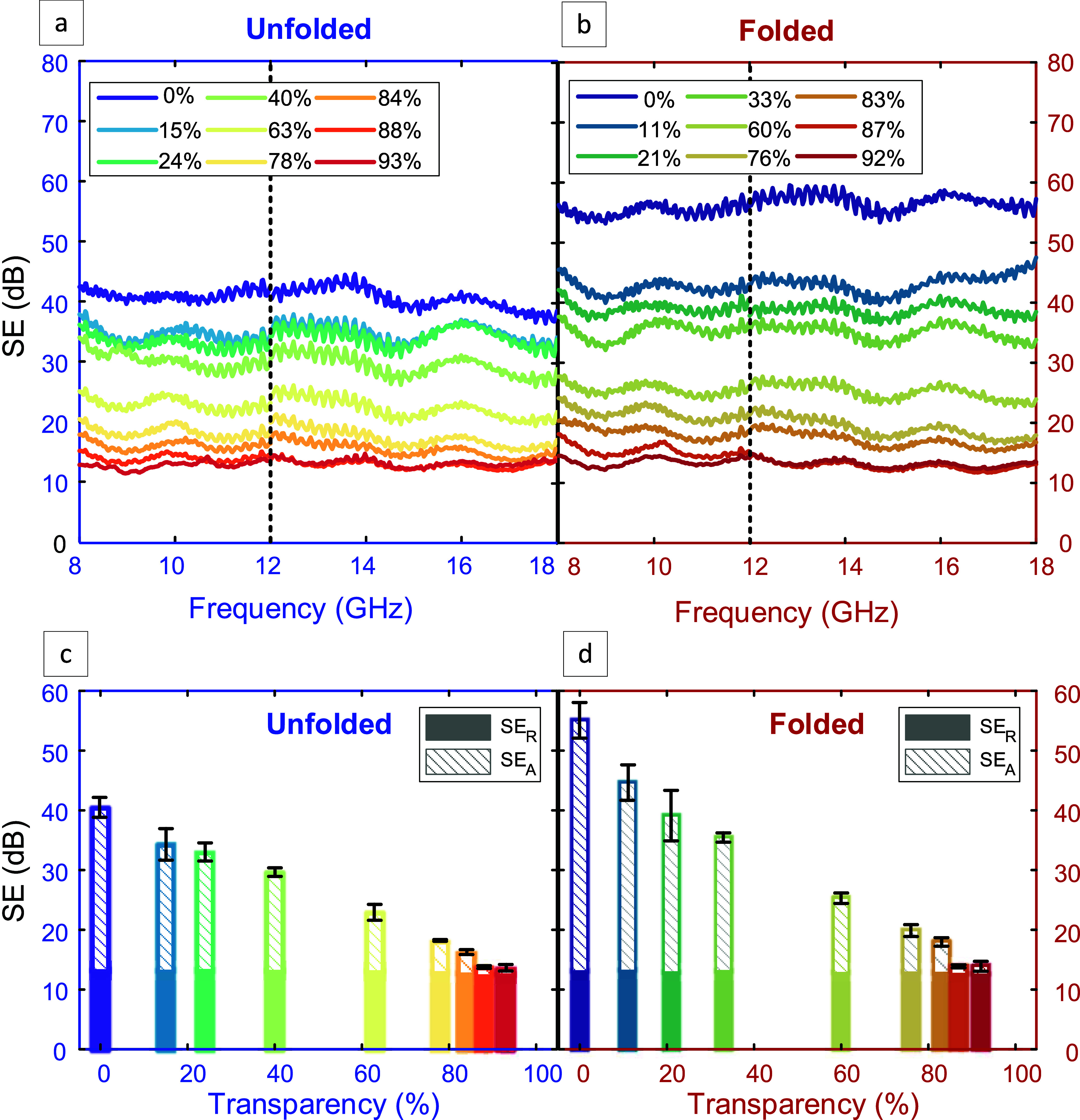
(a) and (b) EMI SE performance of LIG-based samples fabricated with different transparency values (expressed as percentages in the legend) over a frequency range of 8–18 GHz for unfolded and folded samples, respectively, (c) and (d) SE components of reflection and absorption for samples at multiple transparencies for unfolded and folded, respectively. The error bars represent the standard error based on three independent samples (*n* = 3).

In the EMI SE results (Figure 4a, b), the vertical dotted line at 12 GHz marks the point where the flange is changed in the vector network analyzer for subsequent frequency analysis. Our results show that the SE levels decrease with increasing transparency, which is attributed to reduced content of conductive material (LIG) in the higher transparency samples. The measured total EMI SE as a function of sample transparency is included in Figure S7a–c, together with its reflection (SE_R_) and absorption (SE_A_) components, calculated from the measured scattering parameters using eqs 1–([Disp-formula eq6]) in the Experimental Section. As seen in Figure S7, the SE_R_ remains nearly unchanged across different transparencies and between unfolded and folded samples, whereas the SE_A_ increases markedly for the folded designs. Generally, our folded samples show high EMI SE values, exceeding 40 dB for samples with 11% transparency. This superior SE for folded samples is attributed to the stacking of two LIG layers with matching hexagonal geometries without compromising transparency. The slightly lower transparency observed in the folded samples arises from minor geometric misalignment of the honeycomb webs after folding, which marginally reduces the open-area fraction without altering the intrinsic optical properties of LIG. While this small decrease in transparency can contribute to a modest increase in shielding due to greater conductive coverage, the substantial enhancement in SE primarily results from the folded architecture itself, which doubles the conductive pathways and increases internal reflections between the stacked LIG layers.

Figure 4c,d summarize the SE results for all unfolded and folded samples, respectively, ranging from 0% to 95% transparency with their corresponding SE_A_ and SE_R_ components. Additionally, a sample with 100% transparency, essentially a piece of polyimide with a laser-cut square and no LIG was measured to demonstrate the impact of adding the LIG webs on SE. The SE values arising from this 100% transparency sample likely arise from residual conductive carbon on the laser-cut edges. Also, EMI SE for air and for bare polyimide are measured to be about insignificant (below 0.3 dB) in the same frequency range (8–18 GHz), as shown in Figure S8. Taken together with the control measurements on air and bare polyimide (Figure S8), these results confirm that the improvement in SE is mainly due to the absorption of electromagnetic waves (increase in SE_A_), with no noticeable change in the reflection of the waves (SE_R_). This consistency arises because reflection is primarily dictated by the impedance mismatch between air and the conductive LIG surface, which is determined by the surface conductivity and morphology of the outermost layer. Folding increases the overall thickness of the shield but does not modify this interface. Thus, the reflection component remains largely unchanged. Instead, the enhanced total SE of the folded samples originates from an increase in the absorption component, resulting from multiple internal reflections and extended propagation paths within the folded structure. For the same underlying reason, variations in transparency also exhibit nearly similar SE_R_ as the honeycomb sizes are relatively smaller than the wavelengths of the incident radiation. Therefore, within the measured frequency range, the different hexagonal geometries do not influence how the electromagnetic waves reflect from the substrate. Instead, the conductive LIG absorbs the electromagnetic waves, providing protection against interference.

Both unfolded and folded samples achieve robust shielding results, peaking at 40 and 55 dB, respectively. Additionally, samples with over 90% transparency still achieved an SE of 14 dB. A near-linear trend is observed in the drop of EMI SE with increasing transparencies, particularly beyond 40%. For shields with less than 40% transparency, folding increased SE more effectively than for shields with greater transparency. This is attributed to differences in pattern design and manufacturing: sub-40% shields used hexagonal patterns of the same pitch but varying width, whereas those with transparencies above 40% used consistent width but varying pitch. The values of the width and pitch calculated based on the nominal transparencies are illustrated in Table S1. To balance transparency and EMI shielding performance, we systematically tuned the honeycomb pitch and web width to control the open-area fraction (Figure S1), and optimized LIG conductivity by adjusting laser power, speed, defocus, and raster gap, building upon on our recent studies.^58,60,62^ This optimization not only improves the electrical properties of the LIG but also influences its microstructural characteristics, which are critical for electromagnetic attenuation. Beyond achieving low sheet resistance, the conductive and porous microstructure of the LIG facilitates multiple internal scattering of incident electromagnetic waves, thereby enhancing the absorption component of the total shielding. In addition, folding the samples effectively increased the effective thickness of the shields, providing longer propagation paths for wave attenuation. This combined optimization yielded a transparency range of 11–60%, within which the folded shields maintained >20 dB EMI SE, demonstrating an effective trade-off between optical and electrical performance. Overall, our EMI SE results, spanning a wide range of transparencies, illustrate the effectiveness of the LIG-based kirigami design as a transparent EMI shielding solution, relevant for applications such as display windows and wearable electronic devices.

To place our results within the wider field of EMI shielding materials, we first focus on the most commonly used metallic EMI shield. Metals, such as copper and silver, are widely known for their excellent EMI shielding properties due to their high electrical conductivity. However, major downsides, such as high manufacturing costs and density, severely limit the use of these materials in many applications, especially where flexibility and transparency are required. In particular, the use of expensive metals, such as silver, can massively raise production costs, rendering its use prohibitive. Metals are generally dense materials, making them heavy and less suitable for lightweight systems in the aerospace industry or for polymer-based flexible devices.

While metals can be integrated with flexible devices if they are sufficiently thin, their stiffness and thickness greatly limit their flexibility. Figure 5a illustrates the theoretically obtained maximum thicknesses allowed for a material to be considered flexible without crossing the elastic limit. This is based on the calculated radius of curvature (*R*), considering the elastic modulus (*E*), film thickness (*t*) and yield strength (σ_*y*_) (using equation of the bending stress in terms of strain; $\sigma_{y}=E\frac{t}{R}$ ). For example, to achieve the same 30 mm radius of curvature, the allowable thicknesses for metals such as silver and nickel is much lower than that of carbon-based materials as shown in Figure 5c. For manufacturing metals at such low thicknesses (around 10 μm), a variety of complex steps are involved whereas the process for LIG in air is a facile alternative that does not require expensive machines or vacuum system. Moreover, the energy-consuming processes involved in producing and shaping metallic shields typically have a significant carbon footprint,^66^ further highlighting the contrast to LIG.

**Figure 5:**
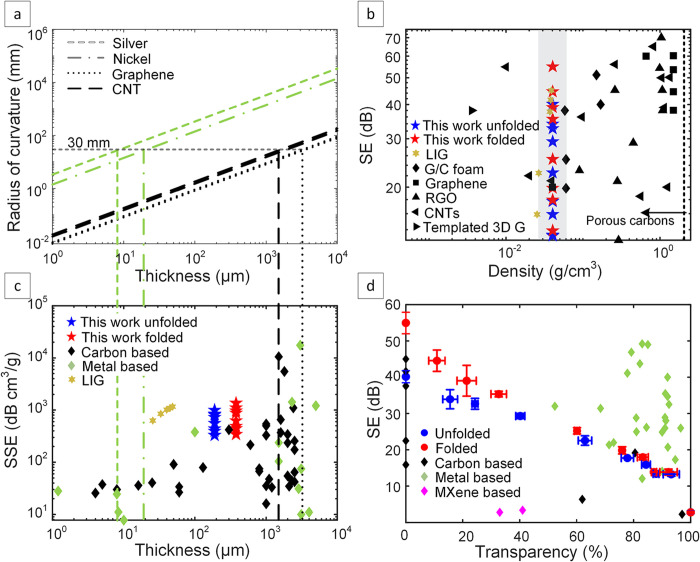
(a) Theoretically maximum thickness based on radius of curvature for different materials to assess the flexibility, (b) SE of various reported studies of carbon based materials compared to this work, (c) SSE of different types of materials reported with their thicknesses compared to this work with limits of thickness to remain flexible and (d) SE of studies on semi transparent materials based on carbon, metal and MXene compared to this work’s unfolded and folded EMI SE shields. The corresponding values (including SE, SSE, thickness, density, and optical transparency at 550 nm) and literature sources for the compared data are summarized in Tables S2 and S3. The error bars represent the standard error based on three independent samples (*n* = 3).

In Figure 5c, the SSE is plotted after normalizing measured SE values to the material density. This facilitates a more accurate comparison taking into consideration both the density and thickness. The LIG layer thickness in the unfolded samples, determined from cross-sectional SEM images, was approximately 190 μm. Because the folded samples were produced using identical laser parameters, their two LIG layers exhibit the same morphology and thickness; therefore, the total effective thickness considered in the normalization was doubled (380 μm). This normalization enables a fair comparison with other reported materials. For example, within the thickness range of 10–100s μm, carbon-based materials are clearly superior to metals, and our results show the best SSE values in this thickness range (The corresponding data and references are summarized in Table S2). Thus, carbon-based alternatives, such as LIG, offer a solution to the issues of flexibility and weight.

Figure 5b maps SE values reported for carbon-based materials having different densities compared to our work (a version with a linear *y*-axis is provided in Figure S14 in the Supporting Information). The black dotted line represents the density of graphene and the materials to the left are porous carbons that include LIG, graphene/carbon foam (G/C foam), graphene, reduced graphene oxide (RGO), CNTs and templated 3D graphene (The corresponding data and references are summarized in Table S2). The shaded region represents the regime of LIG, which shows that our work exhibits the best performance in terms of SE for folded LIG EMI shields with over 50 dB of SE for the low density of 0.04 g/cm^3^. While some porous carbon-based materials, such as templated 3D graphene and vertically aligned CNTs, can exhibit even lower densities, they typically involve complex, time-intensive process steps that limit manufacturing scalability. For example, chemical vapor deposition of vertically aligned carbon nanotubes requires the use of specialized high temperature reactors, high purity gas precursors, and often-expensive metal nanocatalysts.^67–69^ On the other hand, LIG fabrication in air from commercial polymers is rapid, scalable, and cost-effective. Thus, the versatility and customizability of LIG make it more suitable for flexible and transparent EMI shields, especially with the high EMI SE performance, which holds a strong advantage over other carbon-based materials such as CNTs or reduced graphene oxides. Thus, the high electrical conductivity of LIG (resistance per unit length value <25 Ω/mm), combined with the flexibility and low density make them ideal for EMI shielding, and the kirigami approach proposed here adds the advantage of transparency as well.

While there is a trade-off between EMI SE and transparency for our samples, as shown in Figure 5d, they still represent a compelling solution, because they do not have to be too thin. Transparent EMI shields have generally been made from thin materials to increase transparency. For example, achieving transparency in MXene-based EMI shields requires limiting the number of layers, which consequently reduces their SE. While adding layers increases the EMI SE, transparency significantly declines. For example, increasing MXene from a single layer (∼2.3 nm) to nine layers (∼18 nm) enhances EMI SE from 1 to 10 dB, but transparency decreases from 90% to 45%.^15^ Further increasing the thickness (to ∼20 μm) achieves an EMI SE exceeding 70 dB, albeit with drastically reduced transparency. A similar trend occurs in metals, where thicker films lose transparency; for instance, silver films transition from high transparency at a few nanometers to near-opacity at 30 nm.^70^ Hence, our kirigami-based approach is superior, as there is no trade-off between transparency and thickness.

To elucidate the optical behavior underlying the observed transparency trends, UV–Vis spectroscopy was performed on fully covered LIG films. The spectra exhibited negligible transmittance (≈0%) across the visible range (400–800 nm), confirming that the LIG regions are optically opaque (Figure S11­(a)). Accordingly, the measured transparency of the kirigami-patterned EMI shields originates exclusively from the laser-cut regions rather than partial light transmission through the LIG. Owing to the small beam size of the UV–Vis relative to the sample dimensions and pitch, direct photospectrometric measurements of the patterned films would not accurately represent their overall optical transmission, as illustrated in Figure S11b, particularly for geometries exhibiting high transparency. Therefore, an image-based transparency analysis was employed to more reliably quantify the apparent optical transmission over the entire sample area. Since this analysis was conducted under visible illumination (≈400–700 nm), which substantially overlaps with the 550 nm wavelength used in UV–Vis measurements reported in Figure 5d, the comparison is made on a consistent spectral basis (The corresponding data and references are summarized in Table S3). This correspondence further validates the reliability of the image-based transparency quantification and justifies its direct comparison with literature data reported at 550 nm.

## Conclusion

In this study, we have successfully developed flexible, transparent, and lightweight EMI shields by combining laser-based cutting and laser-induced graphitization of polyimide films. Our patterned 3D porous LIG films achieved an exceptional EMI SE surpassing 50 dB at a remarkably low density of 0.04 g/cm^3^, underscoring the advantages of carbon-based materials in delivering both high shielding performance and low weight. Through the incorporation of kirigami, we fine-tuned both the SE and transparency of our graphene-based films, enabling our LIG EMI shields to reach an SE of over 17 dB while maintaining transparency above 80%. This performance surpasses that of existing carbon nanomaterials, while the LIG structure retains better mechanical flexibility compared to conventional metallic shields. Therefore, the integration of LIG and kirigami presents a powerful solution for achieving custom EMI shielding that meets demanding criteria for efficiency, transparency, lightness, and flexibility. Ultimately, our approach leverages rapid, cost-effective, and scalable laser processing of commercial polymers in ambient conditions, providing a practical path to sustainable, high-performance graphene-based EMI shields that offer enhanced shielding capabilities in a direct write one step process.

## Supplementary Material



## Notes

Patterning of hexagonal cuts with pitch, width, and side length parameters to control transparency; image processing example (88% transparency) for assessing EMI shield transparency postfabrication; topographic overhead and isometric views of bare PI and EMI shields with varying gap sizes; comparison of maximum profile heights for bare PI and EMI shields; SEM images of LIG morphology with visible laser paths, cross-sectional views confirming adhesion and thickness; variation of total, reflected, and absorbed EMI SE with transparency for folded and unfolded shields; EMI SE for air and PI over a frequency range from 8–18 GHz; optical microscopy images showing lines used for resistance per unit length measurements with varying laser defocus; nominal and image-processed transparencies in honeycomb patterns, with transparency data pre- and postfabrication (PDF)

## Abbreviations

LIG: laser-induced graphene
EMI: electromagnetic interference
SE: shielding efficiency
SSE: specific shielding efficiency
NWs: nano wires
CNTs: carbon nanotubes
CNFs: carbon nanofibers
PI: polyimide
