# Supplementary material for: Combining Laser-Induced Graphene with Kirigami for Transparent Flexible Electromagnetic Interference Shielding
Source: ACS Appl Eng Mater. 2025 Dec 17;4(2):490–501. doi: 10.1021/acsaenm.5c00861 (PMC12954757; doi:10.1021/acsaenm.5c00861)
Supplement: Supplementary file 1 [file em5c00861_si_001.pdf]

Supporting Information

Combining Laser-Induced Graphene with Kirigami for Transparent Flexible Electromagnetic Interference Shielding

*Mirza Sahaluddin[1], Mingxuan Li[2], Mehdi Zarei[1], Paul W Leu[1,2,3] and Mostafa Bedewy[1,2,3]**

[1] Department of Mechanical Engineering and Materials Science, University of Pittsburgh, 3700 O’Hara Street, Pittsburgh, PA 15261, USA

[2] Department of Chemical and Petroleum Engineering, University of Pittsburgh, 3700 O’Hara Street, Pittsburgh, PA 15261, USA

[3] Department of Industrial Engineering, University of Pittsburgh, 3700 O’Hara Street, Pittsburgh, PA 15261, USA

*author to whom correspondence should be addressed.

E-mail address: mbedewy@pitt.edu (M. Bedewy)

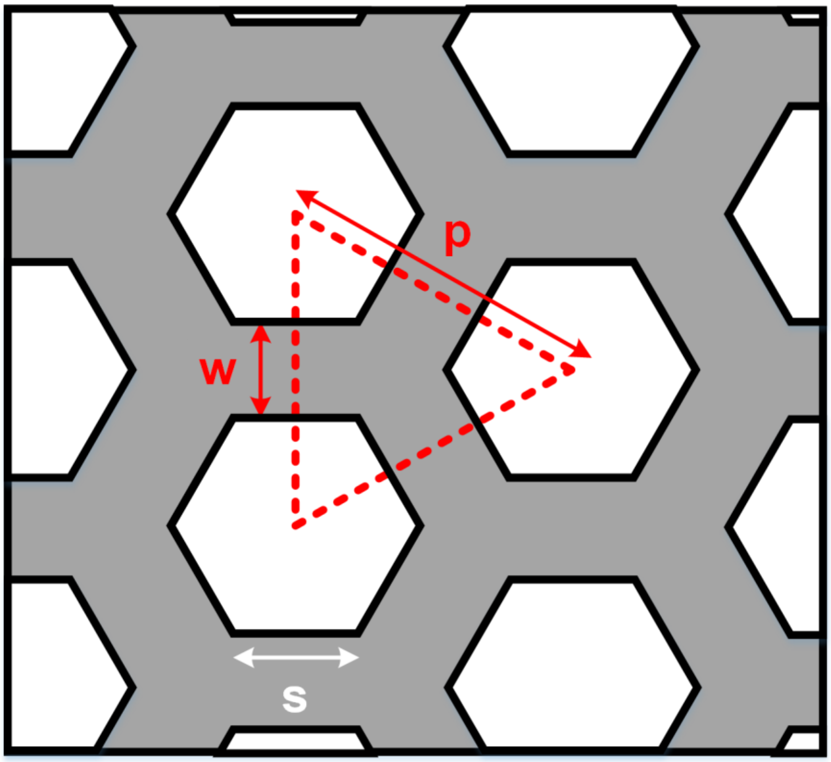

Figure S1: Patterning of hexagonal cuts showing pitch (p) and width (w) of honeycomb pattern (s represents the side length of each hexagon). These geometric parameters are used to design different patterns with varying degrees of transparency, by controlling the area ratio between the cut hexagons and the LIG-covered webs.

The nominal transparency (%) was calculated using the formula:

$$Transparency = \frac{(p-w)^2}{p^2} \tag{S1}$$

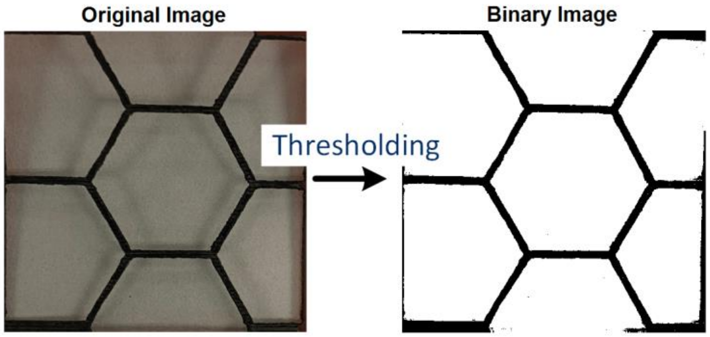

Figure S2: Sample image (88% image processing transparency) demonstrating the image processing of the EMI shields to assess transparency following fabrication. The processing was conducted using MATLAB, as outlined in the experimental section.

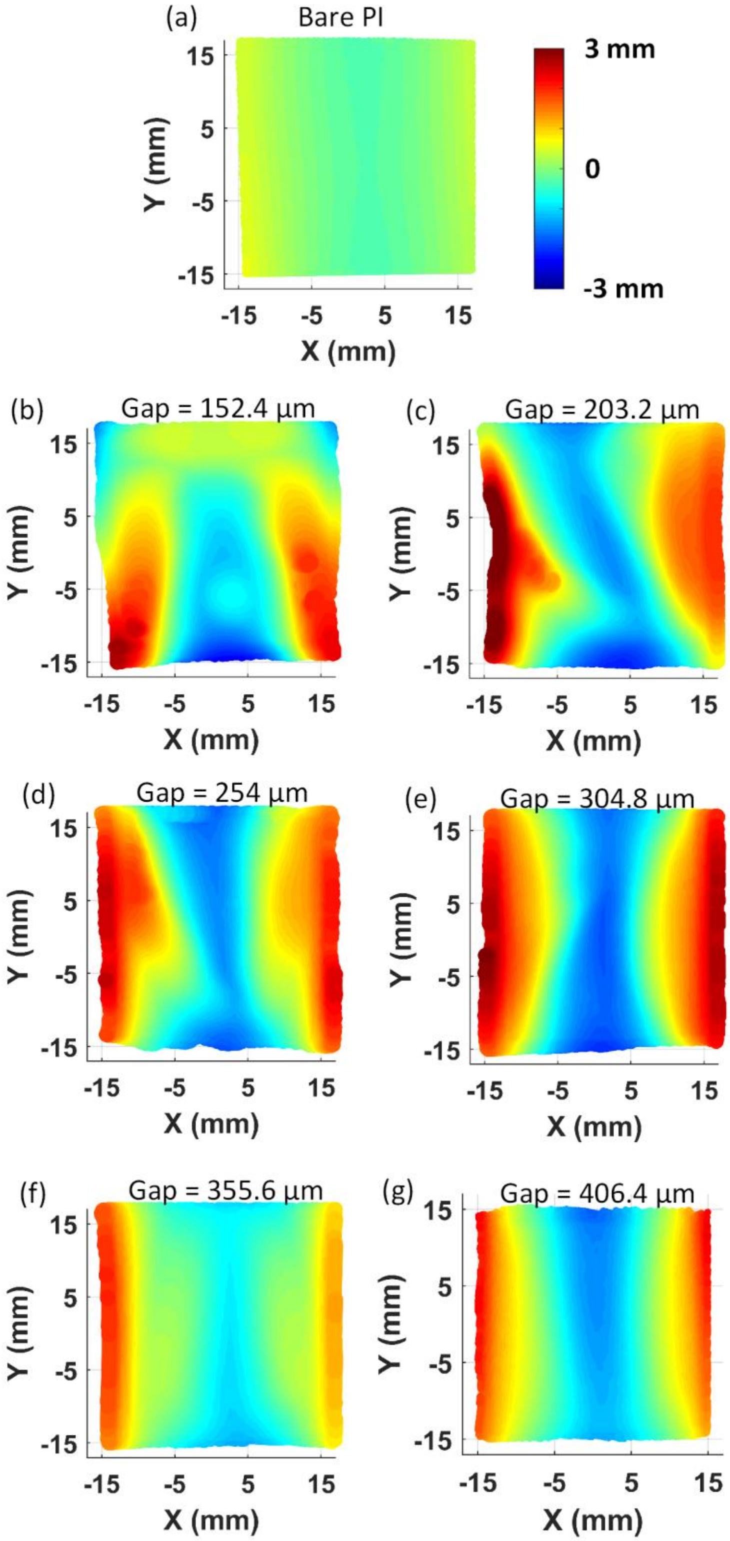

Figure S3: (a) Overhead view of the topography of bare PI (b)-(g) Overhead view of the topography of EMI shields with gaps 0.152mm, 203.2mm, 254mm, 304.8mm, 355.6mm and 406.4mm, respectively. Color bar scale is for all the figures

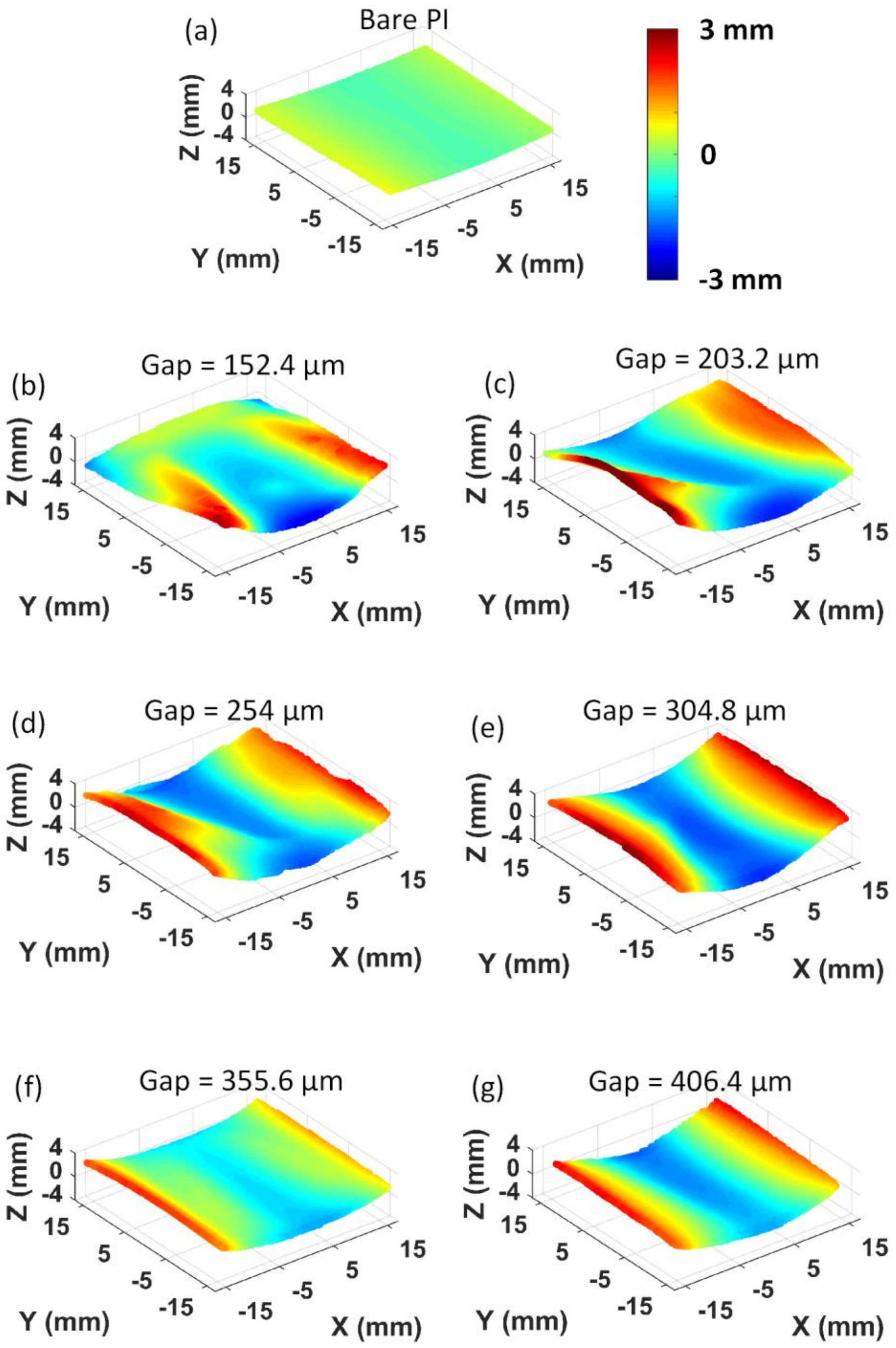

Figure S4: (a) Isometric view of the topography of bare PI (b)-(g) Isometric view of the topography of EMI shields with gaps 0.152mm,203.2mm, 254mm, 304.8mm, 355.6mm and 406.4mm, respectively. Color bar scale is for all the figures

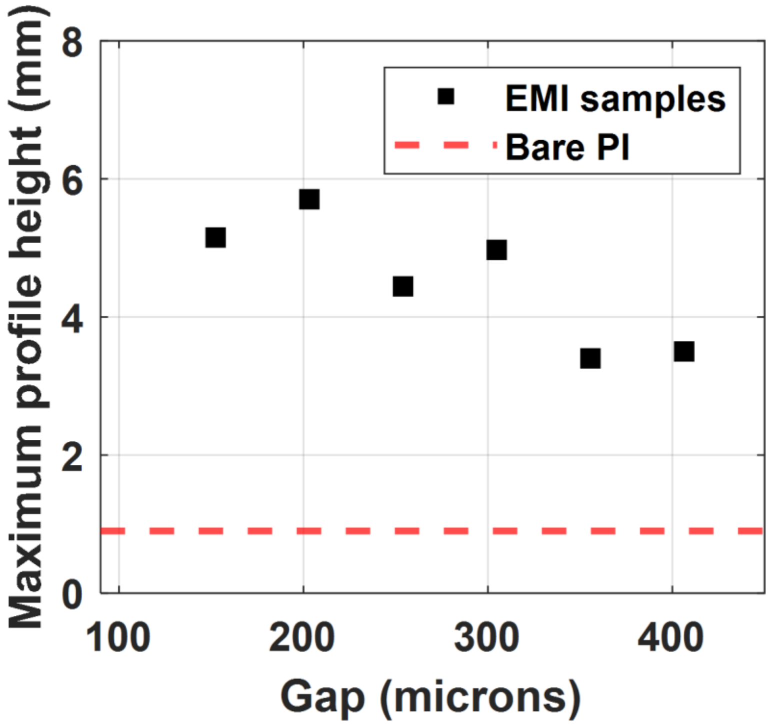

Figure S5: Maximum profile height for bare PI and EMI shields of various gaps

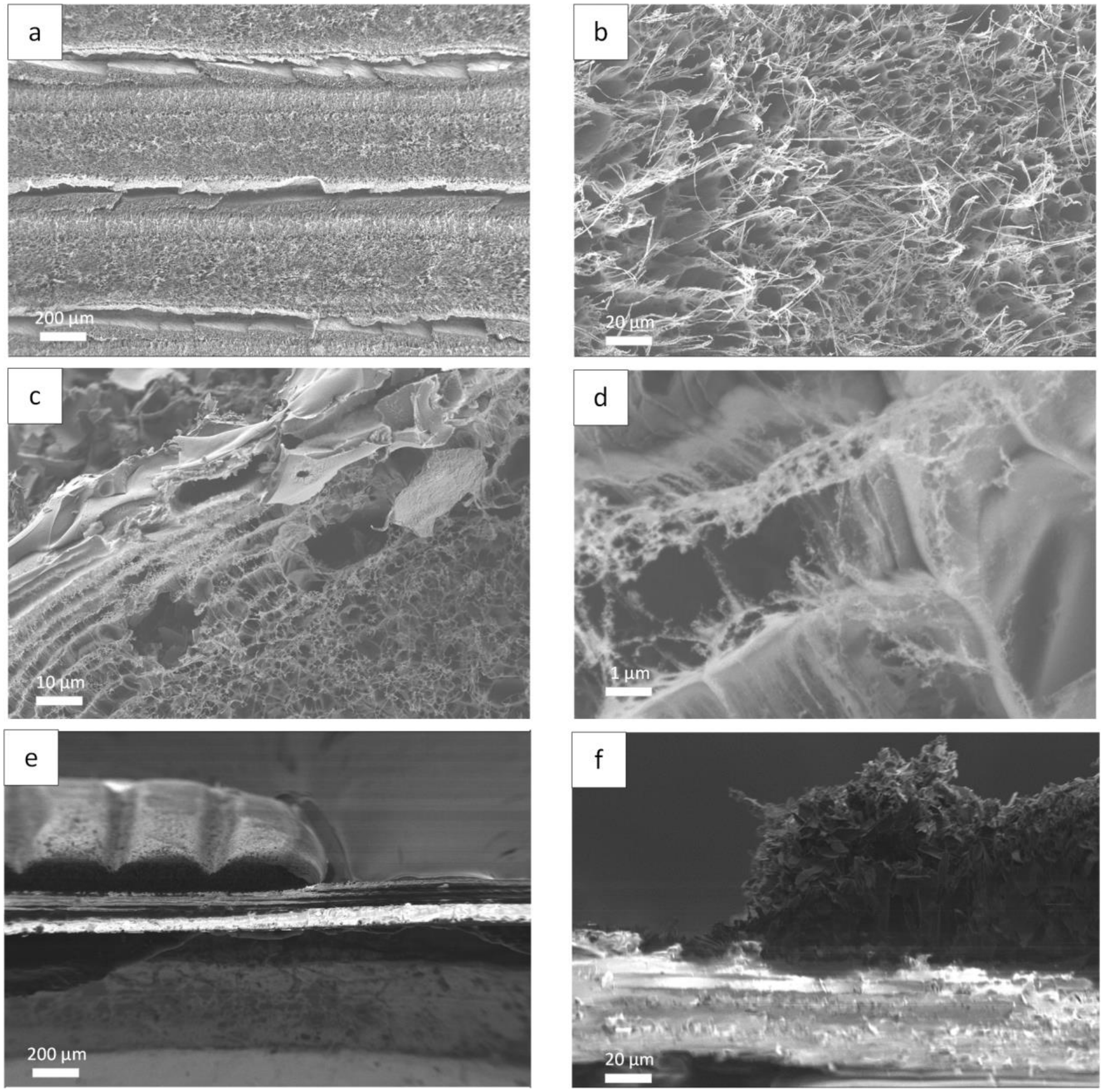

Figure S6: Characterization of the morphology of laser induced graphene (LIG) areas formed using the optimized gap of 355 μm at power 12.5 W, speed 111 mm/s and defocus 6 mm and two passes: (a-b) Top view SEM images showing the porous morphologies of LIG with the laser paths visible, highlighting the continuous nature of the LIG areas obtained by overlapping laser passes in raster mode. (c-d) Transition morphology between the regions near and away from the cut, showing similar porous morphology to the overall SEM top views. (e-f) Side view SEMs showing the

cross-sectional view of our LIG layers, confirming the strong adhesion and estimated thickness values.

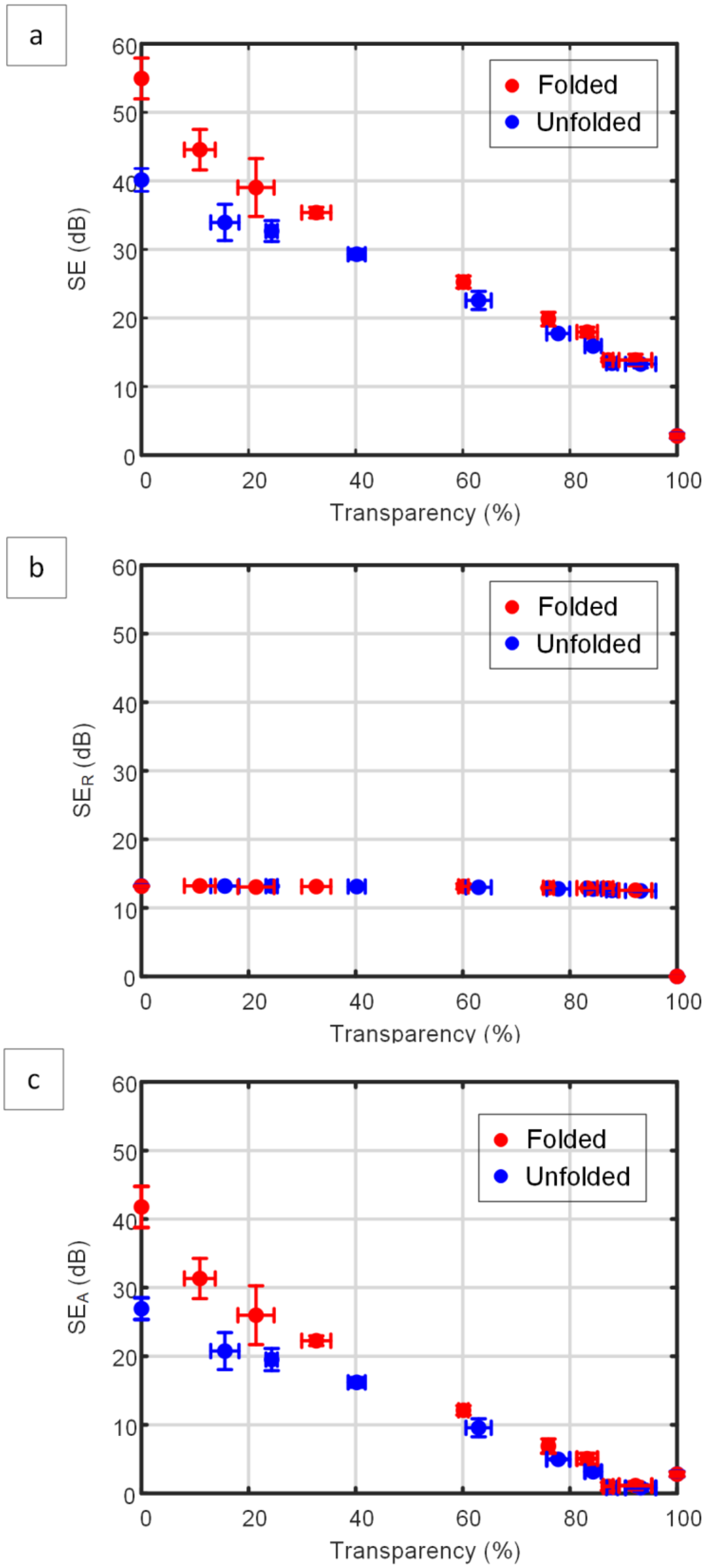

Figure S7: (a) Total EMI SE variation with transparency, (b) Reflected EMI SE variation with transparency and (c) Absorbed EMI SE variation with transparency for folded and unfolded EMI shields

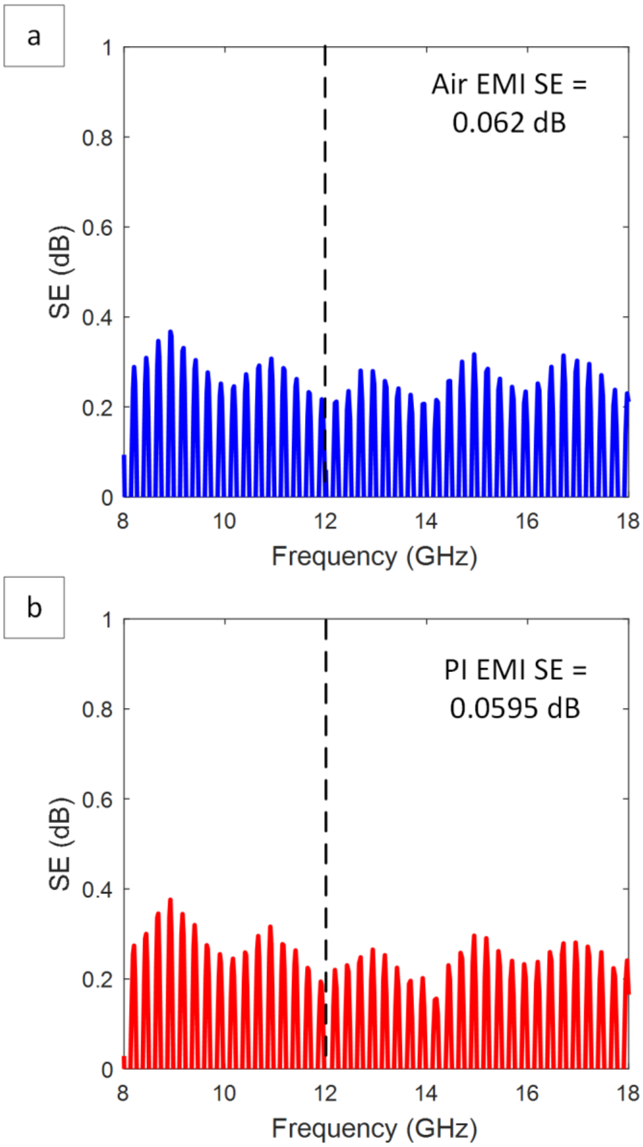

Figure S8: (a) EMI SE for air measured across a frequency range from 8 GHz to 18 GHz and (b) EMI SE for PI measured across a frequency range from 8 GHz to 18 GHz

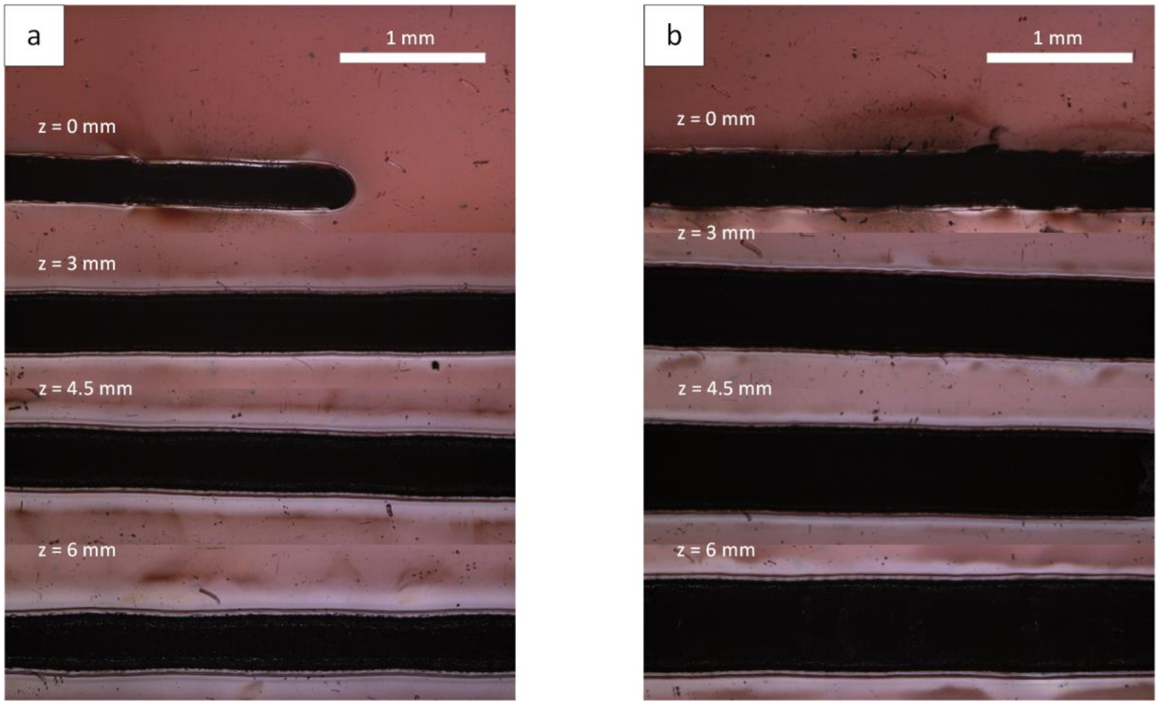

Figure S9: Optical microscopy images of lines at different levels of defocus (z) used to measure resistance per unit length at (a) P = 12.52 W, speed = 111 mm/s at two laser passes and (b) P= 23.2 W, speed = 111 mm/s at two laser passes

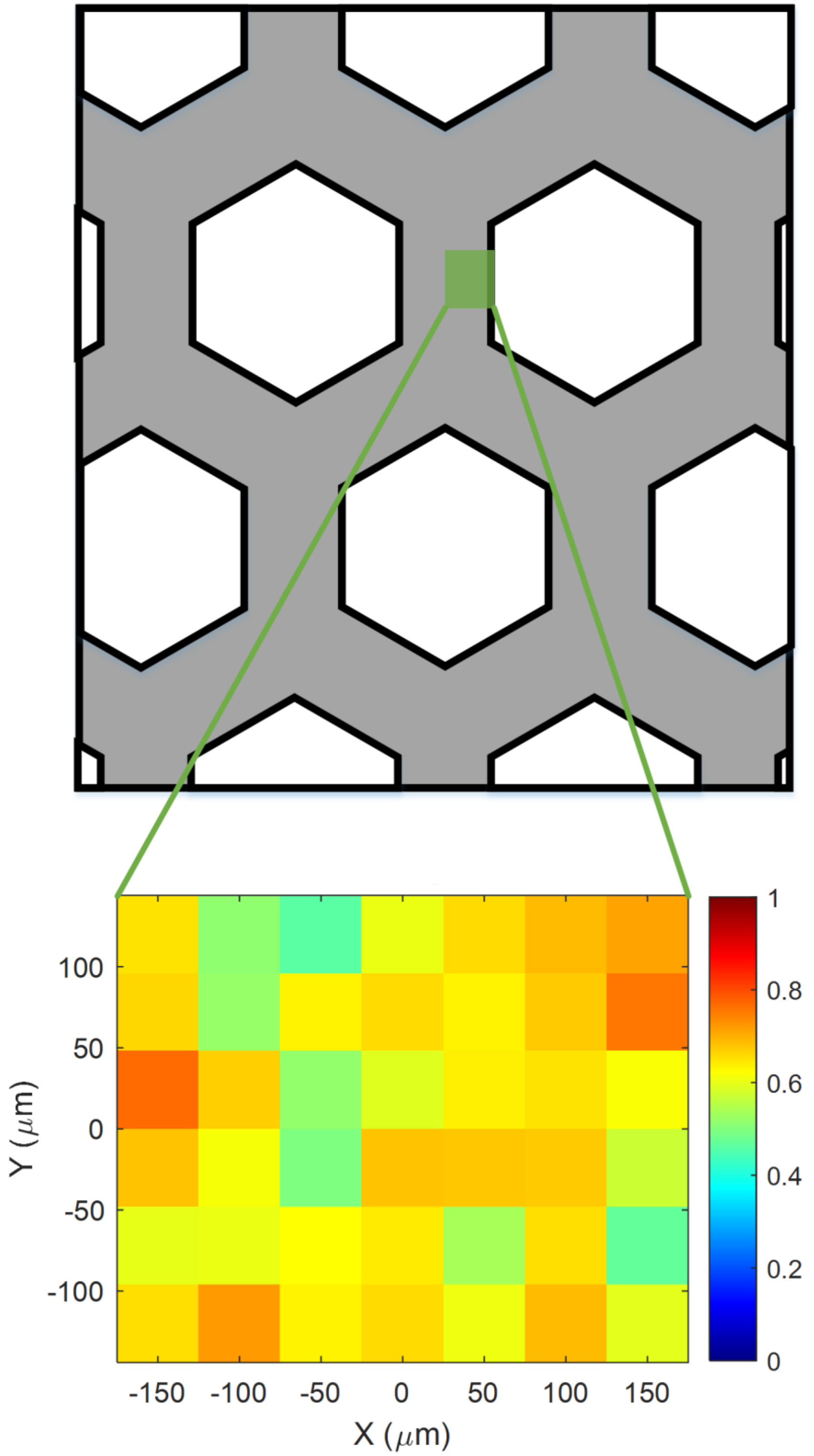

Figure S10: Areal Raman map of the 2D/G intensity ratio illustrating that the graphene quality remain approximately uniform across the scanned region, with similar ratios near the edge and away from the edge. The scale bar represents the 2D/G intensity ratios

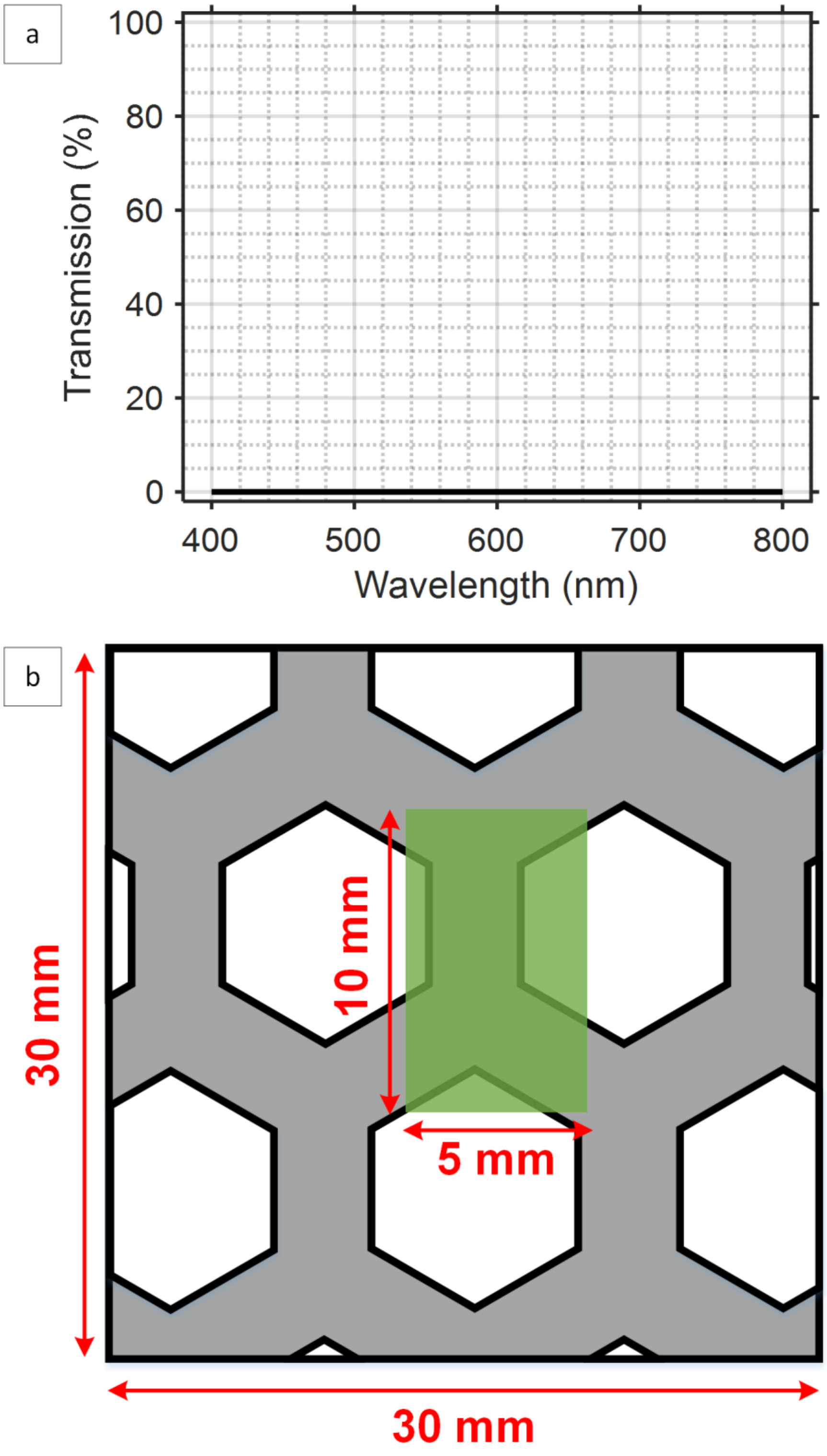

Figure S11: (a) UV–Vis spectrum of a fully covered LIG film confirming its optical opacity (~0% transmittance). (b) Illustration of the beam-size limitation in UV–Vis spectroscopy, which prevents accurate transmittance measurement of patterned transparent samples.

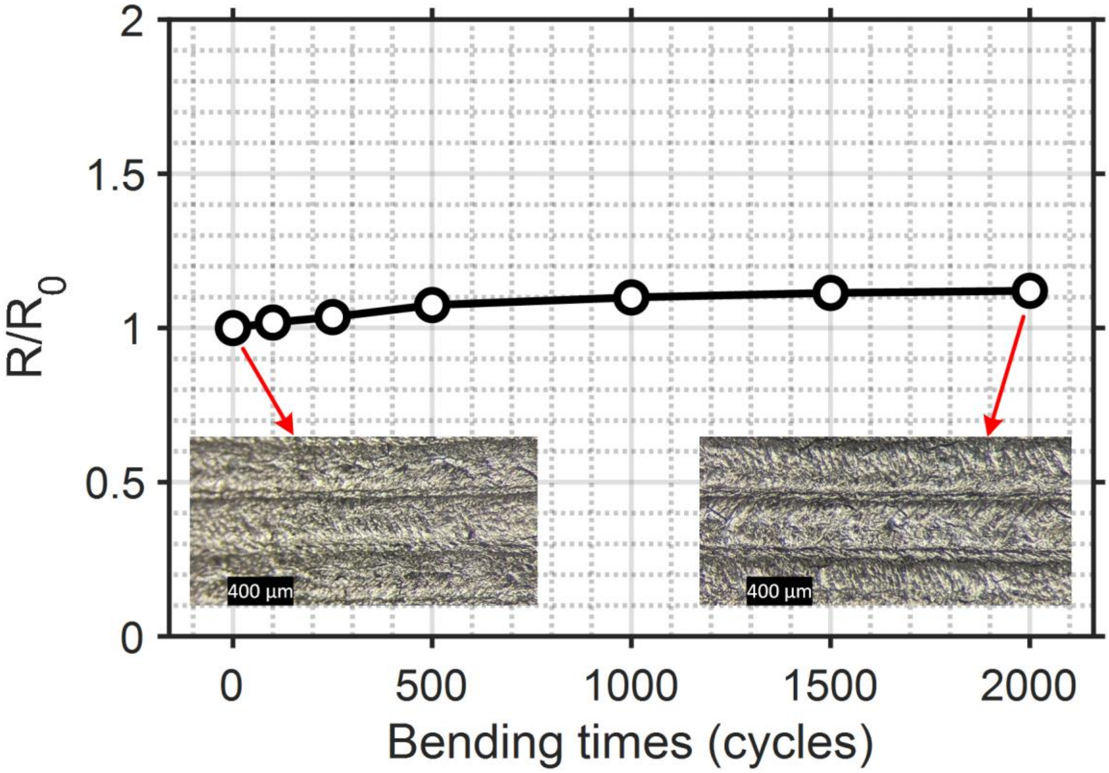

Figure S12: Normalized resistance (R/R_0) as a function of bending cycles. The plot shows the variation in electrical resistance of the sample relative to its initial value (R_0) during repeated bending to a diameter of 8 mm. The fully covered LIG sample exhibits minimal change in resistance (< 11%) even after 2000 cycles. The insets display optical microscopy images of the sample before and after the bending test.

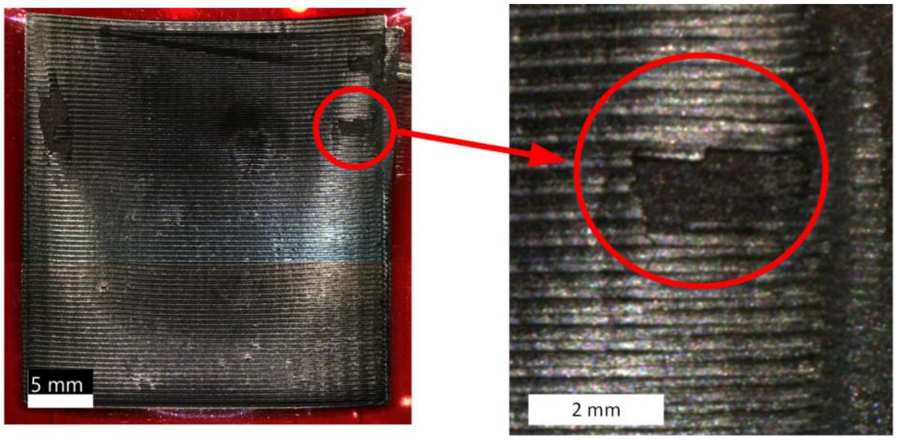

Figure S13: Stitched optical microscopy top-view image of the sample fabricated with the lowest raster gap (0.152 mm), showing localized delamination of the LIG layer.

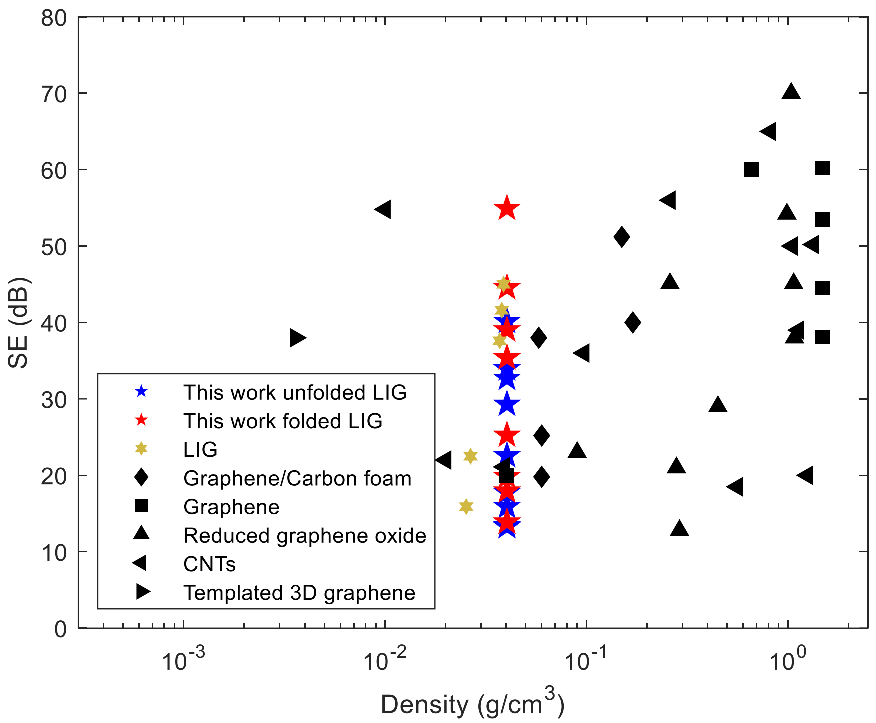

Figure S14: Comparison of EMI SE vs density for various reported carbon-based materials and this work (y-axis plotted on a linear scale).

Table S1: Nominal transparency (pre-fabrication) displaying the pitch and width of the honeycomb pattern, alongside the image processing transparencies (post-fabrication) of the samples. A representative sample of one of the image processing transparencies is illustrated in Figure S2. The nominal transparencies were calculated using equation (1) while image processing transparencies were measured through thresholding in MATLAB, as described in the experimental section.

**Nominal Transparency**			**Image processing transparency**	
**Transparency**	**p (mm)**	**w (mm)**	**Unfolded**	**Folded**
15	3.3	2.02	15.563	10.893
25	3.3	1.65	24.303	21.382
40	3.3	1.2	40.182	32.639
60	3.3	0.75	62.94	60.114
80	7	0.75	77.81	75.973
85	9.5	0.75	84.331	83.213
90	14	0.75	87.859	87.144
95	26.9	0.75	93.196	92.221

Table S2: Summary of thickness, density, EMI shielding effectiveness (SE), and specific shielding effectiveness (SSE) of representative carbon-based and metal-based materials reported in the literature

Type	Thickness (cm)	Density (g/cm^3)	SE (dB)	SSE (dBcm^3/g)	Reference
LIG	0.00255	0.0253	15.9	628.458498	[1]
	0.0033	0.0266	22.5	845.8646617	[1]
	0.004	0.0371	37.6	1013.477089	[1]
	0.00447	0.038	41.6	1094.736842	[1]
	0.00483	0.0388	45	1159.793814	[1]
Reduced graphene oxide (RGO)	0.23	0.29	12.8	44.13793103	[2]
	0.08	1.04	70	67.30769231	[3]
	0.25	0.45	29	64.44444444	[4]
	0.08	0.28	21	75	[5]
	0.2	0.99	54.2	54.74747475	[6]
	0.1	1.08	38	35.18518519	[7]
	0.25	0.26	45.1	173.4615385	[8]
	0.25	1.07	45.1	42.14953271	[9]
	0.25	0.09	23	255.5555556	[10]
Graphene	0.1	0.04	20	500	[11]
	0.005	0.66	60	90.90909091	[12]
	0.0004	1.49	38.1	25.5704698	[13]
	0.0008	1.49	44.5	29.86577181	[13]
	0.0016	1.49	53.5	35.90604027	[13]
	0.0025	1.49	60.2	40.40268456	[13]
Templated 3D graphene	0.15	0.0036	38	10555.55556	[14]
G/C foam	0.03	0.06	25.2	420	[15]
	0.1	0.06	19.8	330	[11]
	0.2	0.15	51.2	341.3333333	[16]
	0.2	0.17	40	235.2941176	[17]
	0.16	0.058	38	655.1724138	[18]
CNTs	0.16	0.097	36	371.1340206	[19]
	0.12	0.56	18.5	33.03571429	[20]
	0.1	0.039	21.1	541.025641	[21]
	0.21	1.13	39	34.51327434	[22]
	0.11	1.05	50	47.61904762	[23]
	0.0005	1.34	50.2	37.46268657	[24]
	0.1	1.25	20	16	[25]
	0.013	0.82	65	79.26829268	[26]
	0.06	0.26	56	215.3846154	[27]
	0.18	0.01	54.8	5480	[28]
	0.24	0.02	22	1100	[29]

Metal based	0.31	9	90	10	[30]
	0.001	9	70	7.777777778	[31]
	0.00012	1.6	44.7	27.9375	[32]
	0.285	1.87	58	31.01604278	[33]
	0.15	0.24	25	104.1666667	[34]
	0.15	0.23	54.6	237.3913043	[34]
	0.0008	2.7	66	24.44444444	[31]
	0.4	8.09	89	11.00123609	[30]
	0.31	0.64	48	75	[33]
	0.5	0.029	35	1206.896552	[35]
	0.3	0.004	70.1	17525	[36]
	0.23	0.045	64	1422.222222	[37]
	0.01	0.2	76	380	[38]
This work - unfolded	0.0189	0.04033	40.13666667	995.2062154	
	0.0189	0.04033	33.93883333	841.5282255	
	0.0189	0.04033	32.68361667	810.4045789	
	0.0189	0.04033	29.30368333	726.5976527	
	0.0189	0.04033	22.5665	559.5462435	
	0.0189	0.04033	17.74511667	439.9979337	
	0.0189	0.04033	15.90983333	394.4912803	
	0.0189	0.04033	13.40185	332.3047359	
	0.0189	0.04033	13.29185167	329.5772791	
This work - folded	0.0378	0.04033	54.93623333	1362.167948	
	0.0378	0.04033	44.5492	1104.61691	
	0.0378	0.04033	39.03102333	967.7913051	
	0.0378	0.04033	35.37583333	877.1592694	
	0.0378	0.04033	25.25041667	626.0951318	
	0.0378	0.04033	19.83971667	491.9344574	
	0.0378	0.04033	17.9506	445.0929829	
	0.0378	0.04033	13.88386667	344.2565501	
	0.0378	0.04033	13.87828333	344.1181089	

Table S3: Summary of EMI shielding effectiveness (SE) and optical transmittance (T_{550}) at 550 nm of various materials measured at the specified frequency range

Materials	T_{550} (%)	Frequency (GHz)	SE (dB)	Reference
Graphene	97	2.2-7	2.27	[39]
Multilayer graphene/PET	80.5	18-26.5	19.14	[40]
PEI/rGO/PEI/rGO/PEI	62	8	6.37	[41]
Ni-Pd CNTs	71.4	8.0 - 13	21.37	[42]
ITO/Cu-doped Ag/ITO	96.5	10	26	[43]
ZnO/Ag/ZnO	91.9	4.0-40	34.7	[44]
ZnO/Ag/ZnO	88.9	4.0-40	40.2-56.3	[44]
PES/Ag NWs/PET	81	8	25	[45]
Ag NWs/PDDA	91.3	8.0-12	28	[46]
Ag NWs/PDMS	92.4	8.2-12.4	32.5	[47]
Ag NWs/PET	91.3	8.2-12.4	28.1	[48]
CA/Ag NWs/PU	92	8.0-12	20.7	[49]
PES/Cu NWs/PET	73	8.0-12	22	[50]
Fe2 O3 -modified Ag NWs/PET	90	8.0-12	24.9	[51]
rGO/Ag NWs	91.1	8.2-12.4	35.5	[52]
MXene-Ag NWs/PVA	52.3	8.2-12.4	32	[53]
MXene/Ag NWs/PET	83	8.2-12.4	49.2	[54]
Ag mesh	94.6	12.0-18	17.3	[55]
Petal-shaped Cu mesh	73.4	12.2	32.1	[56]
Cu mesh/PET	85	18	49	[57]
Cu-Ag mesh	82.2	8	43.7	[58]
Ni-Ag mesh	77.8	8	41.1	[58]
Cu mesh	68.4	2.45	31.4	[59]
Ni mesh/PET/Ni mesh	79.2	8.2	46.9	[60]
Ni patterned mesh	91.9	8.2-12.4	40	[61]
Graphene/Al mesh	91	12	28.91	[62]
Graphene/Ni mesh	83	3	12.1	[63]
Hierarchical graphene/Ag mesh/quartz	90	12.0-18	14.1	[64]
Ag mesh/PE	80.9	8.0-12	28.8	[65]
PDMS/Ag mesh/PE	84.5	8.0-12	26.2	[65]
LIG/PI	/	8.2-12.4	15.9	[1]
LIG/PI	/	8.2-12.4	22.5	[1]
LIG/PI	/	8.2-12.4	37.6	[1]
LIG/PI	/	8.2-12.4	41.6	[1]
LIG/PI	/	8.2-12.4	45	[1]
MXene/SWCNT	41	8.2-12.4	3.39	[66]
Mxene/MWCNT	33	8.2-12.4	2.81	[66]

This work - unfolded	0	8.0-12	40.13666667	
	15	8.0-12	33.93883333	
	24	8.0-12	32.68361667	
	40	8.0-12	29.30368333	
	63	8.0-12	22.5665	
	78	8.0-12	17.74511667	
	84	8.0-12	15.90983333	
	88	8.0-12	13.40185	
	93	8.0-12	13.29185167	
This work - folded	0	8.0-12	54.93623333	
	11	8.0-12	44.5492	
	21	8.0-12	39.03102333	
	33	8.0-12	35.37583333	
	60	8.0-12	25.25041667	
	76	8.0-12	19.83971667	
	83	8.0-12	17.9506	
	87	8.0-12	13.88386667	
	92	8.0-12	13.87828333	

References:

(1) Xu, J.; Li, R.; Ji, S.; Zhao, B.; Cui, T.; Tan, X.; Gou, G.; Jian, J.; Xu, H.; Qiao, Y.; Yang, Y.; Zhang, S.; Ren, T. L. Multifunctional Graphene Microstructures Inspired by Honeycomb for Ultrahigh Performance Electromagnetic Interference Shielding and Wearable Applications. *ACS Nano* **2021**, *15* (5), 8907–8918. https://doi.org/10.1021/ACSNANO.1C01552/ASSET/IMAGES/MEDIUM/NN1C01552_M007.GIF.

(2) Ling, J.; Zhai, W.; Feng, W.; Shen, B.; Zhang, J.; Zheng, W. ge. Facile Preparation of Lightweight Microcellular Polyetherimide/Graphene Composite Foams for Electromagnetic Interference Shielding. *ACS Appl. Mater. Interfaces* **2013**, *5* (7), 2677–

2684. https://doi.org/10.1021/am303289m.

(3) Agnihotri, N.; Chakrabarti, K.; De, A. Highly Efficient Electromagnetic Interference Shielding Using Graphite Nanoplatelet/Poly(3,4-Ethylenedioxythiophene)–Poly(Styrenesulfonate) Composites with Enhanced Thermal Conductivity. *RSC Adv.* **2015**, *5* (54), 43765–43771. https://doi.org/10.1039/C4RA15674A.

(4) Yan, D.-X.; Ren, P.-G.; Pang, H.; Fu, Q.; Yang, M.-B.; Li, Z.-M. Efficient Electromagnetic Interference Shielding of Lightweight Graphene/Polystyrene Composite. *J. Mater. Chem.* **2012**, *22* (36), 18772–18774. https://doi.org/10.1039/C2JM32692B.

(5) Li, Y.; Pei, X.; Shen, B.; Zhai, W.; Zhang, L.; Zheng, W. Polyimide/Graphene Composite Foam Sheets with Ultrahigh Thermostability for Electromagnetic Interference Shielding. *RSC Adv.* **2015**, *5* (31), 24342–24351. https://doi.org/10.1039/C4RA16421K.

(6) Xu, F.; Chen, R.; Lin, Z.; Qin, Y.; Yuan, Y.; Li, Y.; Zhao, X.; Yang, M.; Sun, X.; Wang, S.; Peng, Q.; Li, Y.; He, X. Superflexible Interconnected Graphene Network Nanocomposites for High-Performance Electromagnetic Interference Shielding. *ACS Omega* **2018**, *3* (3), 3599–3607. https://doi.org/10.1021/acsomega.8b00432.

(7) Wu, Y.; Wang, Z.; Liu, X.; Shen, X.; Zheng, Q.; Xue, Q.; Kim, J.-K. Ultralight Graphene Foam/Conductive Polymer Composites for Exceptional Electromagnetic Interference Shielding. *ACS Appl. Mater. Interfaces* **2017**, *9* (10), 9059–9069. https://doi.org/10.1021/acsami.7b01017.

(8) Yan, D.-X.; Pang, H.; Li, B.; Vajtai, R.; Xu, L.; Ren, P.-G.; Wang, J.-H.; Li, Z.-M.

Structured Reduced Graphene Oxide/Polymer Composites for Ultra-Efficient Electromagnetic Interference Shielding. *Adv. Funct. Mater.* **2015**, *25* (4), 559–566. https://doi.org/https://doi.org/10.1002/adfm.201403809.

(9) Gao, Y.; Li, Y.; Kong, X.; Ma, M. Enhanced Mechanical Property of Polyamide-6/Graphite Sheet Composites with Segregated 3D Network Binary Structure for High Thermal Conductivity. *Polymers*. 2023. https://doi.org/10.3390/polym15041041.

(10) Gavgani, J. N.; Adelnia, H.; Zaarei, D.; Moazzami Gudarzi, M. Lightweight Flexible Polyurethane/Reduced Ultralarge Graphene Oxide Composite Foams for Electromagnetic Interference Shielding. *RSC Adv.* **2016**, *6* (33), 27517–27527. https://doi.org/10.1039/C5RA25374H.

(11) Chen, Z.; Xu, C.; Ma, C.; Ren, W.; Cheng, H. M. Lightweight and Flexible Graphene Foam Composites for High-Performance Electromagnetic Interference Shielding. *Adv. Mater.* **2013**, *25* (9), 1296–1300. https://doi.org/10.1002/ADMA.201204196.

(12) Zhang, L.; Alvarez, N. T.; Zhang, M.; Haase, M.; Malik, R.; Mast, D.; Shanov, V. Preparation and Characterization of Graphene Paper for Electromagnetic Interference Shielding. *Carbon N. Y.* **2015**, *82*, 353–359. https://doi.org/https://doi.org/10.1016/j.carbon.2014.10.080.

(13) Wei, Q.; Pei, S.; Qian, X.; Liu, H.; Liu, Z.; Zhang, W.; Zhou, T.; Zhang, Z.; Zhang, X.; Cheng, H. M.; Ren, W. Superhigh Electromagnetic Interference Shielding of Ultrathin Aligned Pristine Graphene Nanosheets Film. *Adv. Mater.* **2020**, *32* (14), 1907411.

https://doi.org/10.1002/ADMA.201907411.

(14) Yin, X.; Li, H.; Han, L.; Meng, J.; Lu, J.; Zhang, L.; Li, W.; Fu, Q.; Li, K.; Song, Q. Lightweight and Flexible 3D Graphene Microtubes Membrane for High-Efficiency Electromagnetic-Interference Shielding. *Chem. Eng. J.* **2020**, *387*, 124025. https://doi.org/https://doi.org/10.1016/j.cej.2020.124025.

(15) Shen, B.; Li, Y.; Yi, D.; Zhai, W.; Wei, X.; Zheng, W. Microcellular Graphene Foam for Improved Broadband Electromagnetic Interference Shielding. *Carbon N. Y.* **2016**, *102*, 154–160. https://doi.org/https://doi.org/10.1016/j.carbon.2016.02.040.

(16) Zhang, L.; Liu, M.; Roy, S.; Chu, E. K.; See, K. Y.; Hu, X. Phthalonitrile-Based Carbon Foam with High Specific Mechanical Strength and Superior Electromagnetic Interference Shielding Performance. *ACS Appl. Mater. Interfaces* **2016**, *8* (11), 7422–7430. https://doi.org/10.1021/acsami.5b12072.

(17) Moglie, F.; Micheli, D.; Laurenzi, S.; Marchetti, M.; Mariani Primiani, V. Electromagnetic Shielding Performance of Carbon Foams. *Carbon N. Y.* **2012**, *50* (5), 1972–1980. https://doi.org/https://doi.org/10.1016/j.carbon.2011.12.053.

(18) Kong, L.; Yin, X.; Han, M.; Yuan, X.; Hou, Z.; Ye, F.; Zhang, L.; Cheng, L.; Xu, Z.; Huang, J. Macroscopic Bioinspired Graphene Sponge Modified with In-Situ Grown Carbon Nanowires and Its Electromagnetic Properties. *Carbon N. Y.* **2017**, *111*, 94–102. https://doi.org/https://doi.org/10.1016/j.carbon.2016.09.066.

(19) Song, Q.; Ye, F.; Yin, X.; Li, W.; Li, H.; Liu, Y.; Li, K.; Xie, K.; Li, X.; Fu, Q.; Cheng, L.;

Zhang, L.; Wei, B. Carbon Nanotube–Multilayered Graphene Edge Plane Core–Shell Hybrid Foams for Ultrahigh-Performance Electromagnetic-Interference Shielding. *Adv. Mater.* **2017**, *29* (31), 1701583. https://doi.org/https://doi.org/10.1002/adma.201701583.

(20) Yang, Y.; Gupta, M. C.; Dudley, K. L.; Lawrence, R. W. Novel Carbon Nanotube−Polystyrene Foam Composites for Electromagnetic Interference Shielding. *Nano Lett.* **2005**, *5* (11), 2131–2134. https://doi.org/10.1021/nl051375r.

(21) Zeng, Z.; Jin, H.; Chen, M.; Li, W.; Zhou, L.; Zhang, Z. Lightweight and Anisotropic Porous MWCNT/WPU Composites for Ultrahigh Performance Electromagnetic Interference Shielding. *Adv. Funct. Mater.* **2016**, *26* (2), 303–310. https://doi.org/https://doi.org/10.1002/adfm.201503579.

(22) Pande, S.; Chaudhary, A.; Patel, D.; Singh, B. P.; Mathur, R. B. Mechanical and Electrical Properties of Multiwall Carbon Nanotube/Polycarbonate Composites for Electrostatic Discharge and Electromagnetic Interference Shielding Applications. *RSC Adv.* **2014**, *4* (27), 13839–13849. https://doi.org/10.1039/C3RA47387B.

(23) Al-Saleh, M. H.; Saadeh, W. H.; Sundararaj, U. EMI Shielding Effectiveness of Carbon Based Nanostructured Polymeric Materials: A Comparative Study. *Carbon N. Y.* **2013**, *60*, 146–156. https://doi.org/https://doi.org/10.1016/j.carbon.2013.04.008.

(24) Li, H.; Lu, X.; Yuan, D.; Sun, J.; Erden, F.; Wang, F.; He, C. Lightweight Flexible Carbon Nanotube/Polyaniline Films with Outstanding EMI Shielding Properties. *J. Mater. Chem. C* **2017**, *5* (34), 8694–8698. https://doi.org/10.1039/C7TC02394D.

(25) Li, N.; Huang, Y.; Du, F.; He, X.; Lin, X.; Gao, H.; Ma, Y.; Li, F.; Chen, Y.; Eklund, P. C. Electromagnetic Interference (EMI) Shielding of Single-Walled Carbon Nanotube Epoxy Composites. *Nano Lett.* **2006**, *6* (6), 1141–1145. https://doi.org/10.1021/nl0602589.

(26) Lu, S.; Shao, J.; Ma, K.; Chen, D.; Wang, X.; Zhang, L.; Meng, Q.; Ma, J. Flexible, Mechanically Resilient Carbon Nanotube Composite Films for High-Efficiency Electromagnetic Interference Shielding. *Carbon N. Y.* **2018**, *136*, 387–394. https://doi.org/https://doi.org/10.1016/j.carbon.2018.04.086.

(27) Chaudhary, A.; Kumari, S.; Kumar, R.; Teotia, S.; Singh, B. P.; Singh, A. P.; Dhawan, S. K.; Dhakate, S. R. Lightweight and Easily Foldable MCMB-MWCNTs Composite Paper with Exceptional Electromagnetic Interference Shielding. *ACS Appl. Mater. Interfaces* **2016**, *8* (16), 10600–10608. https://doi.org/10.1021/acsami.5b12334.

(28) Lu, D.; Mo, Z.; Liang, B.; Yang, L.; He, Z.; Zhu, H.; Tang, Z.; Gui, X. Flexible, Lightweight Carbon Nanotube Sponges and Composites for High-Performance Electromagnetic Interference Shielding. *Carbon N. Y.* **2018**, *133*, 457–463. https://doi.org/https://doi.org/10.1016/j.carbon.2018.03.061.

(29) Crespo, M.; González, M.; Elías, A. L.; Pulickal Rajukumar, L.; Baselga, J.; Terrones, M.; Pozuelo, J. Ultra-Light Carbon Nanotube Sponge as an Efficient Electromagnetic Shielding Material in the GHz Range. *Phys. status solidi – Rapid Res. Lett.* **2014**, *8* (8), 698–704. https://doi.org/https://doi.org/10.1002/pssr.201409151.

(30) Ameli, A.; Nofar, M.; Wang, S.; Park, C. B. Lightweight Polypropylene/Stainless-Steel

Fiber Composite Foams with Low Percolation for Efficient Electromagnetic Interference Shielding. *ACS Appl. Mater. Interfaces* **2014**, *6* (14), 11091–11100. https://doi.org/10.1021/am500445g.

(31) Shahzad, F.; Alhabeb, M.; Hatter, C. B.; Anasori, B.; Hong, S. M.; Koo, C. M.; Gogotsi, Y. Electromagnetic Interference Shielding with 2D Transition Metal Carbides (MXenes). *Science (80-.).* **2016**, *353* (6304), 1137–1140. https://doi.org/10.1126/SCIENCE.AAG2421/SUPPL_FILE/SHAHZAD.SM.PDF.

(32) Zeng, Z.; Jiang, F.; Yue, Y.; Han, D.; Lin, L.; Zhao, S.; Zhao, Y.-B.; Pan, Z.; Li, C.; Nyström, G.; Wang, J. Flexible and Ultrathin Waterproof Cellular Membranes Based on High-Conjunction Metal-Wrapped Polymer Nanofibers for Electromagnetic Interference Shielding. *Adv. Mater.* **2020**, *32* (19), 1908496. https://doi.org/https://doi.org/10.1002/adma.201908496.

(33) Shui, X.; Chung, D. D. L. Nickel Filament Polymer-Matrix Composites with Low Surface Impedance and High Electromagnetic Interference Shielding Effectiveness. *J. Electron. Mater.* **1997**, *26* (8), 928–934. https://doi.org/10.1007/s11664-997-0276-4.

(34) Ji, K.; Zhao, H.; Zhang, J.; Chen, J.; Dai, Z. Fabrication and Electromagnetic Interference Shielding Performance of Open-Cell Foam of a Cu–Ni Alloy Integrated with CNTs. *Appl. Surf. Sci.* **2014**, *311*, 351–356. https://doi.org/https://doi.org/10.1016/j.apsusc.2014.05.067.

(35) Ma, J.; Wang, K.; Zhan, M. A Comparative Study of Structure and Electromagnetic Interference Shielding Performance for Silver Nanostructure Hybrid Polyimide Foams. *RSC*

Adv. **2015**, *5* (80), 65283–65296. https://doi.org/10.1039/C5RA09507G.

(36) Wan, Y.-J.; Zhu, P.-L.; Yu, S.-H.; Sun, R.; Wong, C.-P.; Liao, W.-H. Anticorrosive, Ultralight, and Flexible Carbon-Wrapped Metallic Nanowire Hybrid Sponges for Highly Efficient Electromagnetic Interference Shielding. *Small* **2018**, *14* (27), 1800534. https://doi.org/https://doi.org/10.1002/smll.201800534.

(37) Zeng, Z.; Chen, M.; Pei, Y.; Seyed Shahabadi, S. I.; Che, B.; Wang, P.; Lu, X. Ultralight and Flexible Polyurethane/Silver Nanowire Nanocomposites with Unidirectional Pores for Highly Effective Electromagnetic Shielding. *ACS Appl. Mater. Interfaces* **2017**, *9* (37), 32211–32219. https://doi.org/10.1021/acsami.7b07643.

(38) Yu, Q.; Wang, D. Room-Temperature Magnetism in Two-Dimensional Metal–Organic Frameworks Enabled by Electrostatic Gating. *J. Mater. Chem. A* **2023**, *11* (11), 5548–5558. https://doi.org/10.1039/D2TA08540B.

(39) Hong, S. K.; Kim, K. Y.; Kim, T. Y.; Kim, J. H.; Park, S. W.; Kim, J. H.; Cho, B. J. Electromagnetic Interference Shielding Effectiveness of Monolayer Graphene. *Nanotechnology* **2012**, *23* (45), 455704. https://doi.org/10.1088/0957-4484/23/45/455704.

(40) Lu, Z.; Ma, L.; Tan, J.; Wang, H.; Ding, X. Transparent Multi-Layer Graphene/Polyethylene Terephthalate Structures with Excellent Microwave Absorption and Electromagnetic Interference Shielding Performance. *Nanoscale* **2016**, *8* (37), 16684–16693. https://doi.org/10.1039/C6NR02619B.

(41) Kim, S.; Oh, J.-S.; Kim, M.-G.; Jang, W.; Wang, M.; Kim, Y.; Seo, H. W.; Kim, Y. C.; Lee,

J.-H.; Lee, Y.; Nam, J.-D. Electromagnetic Interference (EMI) Transparent Shielding of Reduced Graphene Oxide (RGO) Interleaved Structure Fabricated by Electrophoretic Deposition. *ACS Appl. Mater. Interfaces* **2014**, *6* (20), 17647–17653. https://doi.org/10.1021/am503893v.

(42) Park, J.-B.; Rho, H.; Cha, A.-N.; Bae, H.; Lee, S. H.; Ryu, S.-W.; Jeong, T.; Ha, J.-S. Transparent Carbon Nanotube Web Structures with Ni-Pd Nanoparticles for Electromagnetic Interference (EMI) Shielding of Advanced Display Devices. *Appl. Surf. Sci.* **2020**, *516*, 145745. https://doi.org/https://doi.org/10.1016/j.apsusc.2020.145745.

(43) Wang, H.; Ji, C.; Zhang, C.; Zhang, Y.; Zhang, Z.; Lu, Z.; Tan, J.; Guo, L. J. Highly Transparent and Broadband Electromagnetic Interference Shielding Based on Ultrathin Doped Ag and Conducting Oxides Hybrid Film Structures. *ACS Appl. Mater. Interfaces* **2019**, *11* (12), 11782–11791. https://doi.org/10.1021/acsami.9b00716.

(44) Yuan, C.; Huang, J.; Dong, Y.; Huang, X.; Lu, Y.; Li, J.; Tian, T.; Liu, W.; Song, W. Record-High Transparent Electromagnetic Interference Shielding Achieved by Simultaneous Microwave Fabry–Pérot Interference and Optical Antireflection. *ACS Appl. Mater. Interfaces* **2020**, *12* (23), 26659–26669. https://doi.org/10.1021/acsami.0c05334.

(45) Hu, M.; Gao, J.; Dong, Y.; Li, K.; Shan, G.; Yang, S.; Li, R. K.-Y. Flexible Transparent PES/Silver Nanowires/PET Sandwich-Structured Film for High-Efficiency Electromagnetic Interference Shielding. *Langmuir* **2012**, *28* (18), 7101–7106. https://doi.org/10.1021/la300720y.

(46) Zhu, X.; Xu, J.; Qin, F.; Yan, Z.; Guo, A.; Kan, C. Highly Efficient and Stable Transparent Electromagnetic Interference Shielding Films Based on Silver Nanowires. *Nanoscale* **2020**, *12* (27), 14589–14597. https://doi.org/10.1039/D0NR03790G.

(47) Jung, J.; Lee, H.; Ha, I.; Cho, H.; Kim, K. K.; Kwon, J.; Won, P.; Hong, S.; Ko, S. H. Highly Stretchable and Transparent Electromagnetic Interference Shielding Film Based on Silver Nanowire Percolation Network for Wearable Electronics Applications. *ACS Appl. Mater. Interfaces* **2017**, *9* (51), 44609–44616. https://doi.org/10.1021/acsami.7b14626.

(48) Yang, H.; Bai, S.; Guo, X.; Wang, H. Robust and Smooth UV-Curable Layer Overcoated AgNW Flexible Transparent Conductor for EMI Shielding and Film Heater. *Appl. Surf. Sci.* **2019**, *483*, 888–894. https://doi.org/https://doi.org/10.1016/j.apsusc.2019.04.034.

(49) Jia, L.-C.; Yan, D.-X.; Liu, X.; Ma, R.; Wu, H.-Y.; Li, Z.-M. Highly Efficient and Reliable Transparent Electromagnetic Interference Shielding Film. *ACS Appl. Mater. Interfaces* **2018**, *10* (14), 11941–11949. https://doi.org/10.1021/acsami.8b00492.

(50) Xie, Q.; Yan, Z.; Wang, S.; Wang, Y.; Mei, L.; Qin, F.; Jiang, R. Transparent, Flexible, and Stable Polyethersulfone/Copper-Nanowires/Polyethylene Terephthalate Sandwich-Structured Films for High-Performance Electromagnetic Interference Shielding. *Adv. Eng. Mater.* **2021**, *23* (8), 2100283. https://doi.org/https://doi.org/10.1002/adem.202100283.

(51) Wang, Z.; Jiao, B.; Qing, Y.; Nan, H.; Huang, L.; Wei, W.; Peng, Y.; Yuan, F.; Dong, H.; Hou, X.; Wu, Z. Flexible and Transparent Ferroferric Oxide-Modified Silver Nanowire Film for Efficient Electromagnetic Interference Shielding. *ACS Appl. Mater. Interfaces*

2020, *12* (2), 2826–2834. https://doi.org/10.1021/acsami.9b17513.

(52) Yang, Y.; Chen, S.; Li, W.; Li, P.; Ma, J.; Li, B.; Zhao, X.; Ju, Z.; Chang, H.; Xiao, L.; Xu, H.; Liu, Y. Reduced Graphene Oxide Conformally Wrapped Silver Nanowire Networks for Flexible Transparent Heating and Electromagnetic Interference Shielding. *ACS Nano* **2020**, *14* (7), 8754–8765. https://doi.org/10.1021/acsnano.0c03337.

(53) Zhou, B.; Su, M.; Yang, D.; Han, G.; Feng, Y.; Wang, B.; Ma, J.; Ma, J.; Liu, C.; Shen, C. Flexible MXene/Silver Nanowire-Based Transparent Conductive Film with Electromagnetic Interference Shielding and Electro-Photo-Thermal Performance. *ACS Appl. Mater. Interfaces* **2020**, *12* (36), 40859–40869. https://doi.org/10.1021/acsami.0c09020.

(54) Chen, W.; Liu, L.-X.; Zhang, H.-B.; Yu, Z.-Z. Flexible, Transparent, and Conductive Ti3C2Tx MXene–Silver Nanowire Films with Smart Acoustic Sensitivity for High-Performance Electromagnetic Interference Shielding. *ACS Nano* **2020**, *14* (12), 16643–16653. https://doi.org/10.1021/acsnano.0c01635.

(55) Zhong, H.; Han, Y.; Lin, J.; Jin, P. Pattern Randomization: An Efficient Way to Design High-Performance Metallic Meshes with Uniform Stray Light for EMI Shielding. *Opt. Express* **2020**, *28* (5), 7008–7017. https://doi.org/10.1364/OE.386921.

(56) Wang, W.; Bai, B.; Zhou, Q.; Ni, K.; Lin, H. Petal-Shaped Metallic Mesh with High Electromagnetic Shielding Efficiency and Smoothed Uniform Diffraction. *Opt. Mater. Express* **2018**, *8* (11), 3485–3493. https://doi.org/10.1364/OME.8.003485.

(57) Walia, S.; Singh, A. K.; Rao, V. S. G.; Bose, S.; Kulkarni, G. U. Metal Mesh-Based Transparent Electrodes as High-Performance EMI Shields. *Bull. Mater. Sci.* **2020**, *43* (1), 187. https://doi.org/10.1007/s12034-020-02159-7.

(58) Voronin, A. S.; Fadeev, Y. V.; Govorun, I. V.; Podshivalov, I. V.; Simunin, M. M.; Tambasov, I. A.; Karpova, D. V.; Smolyarova, T. E.; Lukyanenko, A. V.; Karacharov, A. A.; Nemtsev, I. V.; Khartov, S. V. Cu–Ag and Ni–Ag Meshes Based on Cracked Template as Efficient Transparent Electromagnetic Shielding Coating with Excellent Mechanical Performance. *J. Mater. Sci.* **2021**, *56* (26), 14741–14762. https://doi.org/10.1007/S10853-021-06206-4/METRICS.

(59) Tung, P. D.; Jung, C. W. High Optical Visibility and Shielding Effectiveness Metal Mesh Film for Microwave Oven Application. *IEEE Trans. Electromagn. Compat.* **2020**, *62* (4), 1076–1081. https://doi.org/10.1109/TEMC.2019.2927923.

(60) Jiang, Z.; Zhao, S.; Huang, W.; Chen, L.; Liu, Y. Embedded Flexible and Transparent Double-Layer Nickel-Mesh for High Shielding Efficiency. *Opt. Express* **2020**, *28* (18), 26531–26542. https://doi.org/10.1364/OE.401543.

(61) Jiang, Z.; Huang, W.; Chen, L.; Liu, Y. Ultrathin, Lightweight, and Freestanding Metallic Mesh for Transparent Electromagnetic Interference Shielding. *Opt. Express* **2019**, *27* (17), 24194–24206. https://doi.org/10.1364/OE.27.024194.

(62) Ma, L.; Lu, Z.; Tan, J.; Liu, J.; Ding, X.; Black, N.; Li, T.; Gallop, J.; Hao, L. Transparent Conducting Graphene Hybrid Films To Improve Electromagnetic Interference (EMI)

Shielding Performance of Graphene. *ACS Appl. Mater. Interfaces* **2017**, *9* (39), 34221–34229. https://doi.org/10.1021/acsami.7b09372.

(63) Tran, V. V.; Nguyen, D. D.; Nguyen, A. T.; Hofmann, M.; Hsieh, Y.-P.; Kan, H.-C.; Hsu, C.-C. Electromagnetic Interference Shielding by Transparent Graphene/Nickel Mesh Films. *ACS Appl. Nano Mater.* **2020**, *3* (8), 7474–7481. https://doi.org/10.1021/acsanm.0c01076.

(64) Han, Y.; Liu, Y.; Han, L.; Lin, J.; Jin, P. High-Performance Hierarchical Graphene/Metal-Mesh Film for Optically Transparent Electromagnetic Interference Shielding. *Carbon N. Y.* **2017**, *115*, 34–42. https://doi.org/https://doi.org/10.1016/j.carbon.2016.12.092.

(65) Lei, Q.; Luo, Z.; Zheng, X.; Lu, N.; Zhang, Y.; Huang, J.; Yang, L.; Yang, L.; Gao, S.; Gao, S.; Liang, Y.; He, S.; He, S.; He, S.; He, S. Broadband Transparent and Flexible Silver Mesh for Efficient Electromagnetic Interference Shielding and High-Quality Free-Space Optical Communication. *Opt. Mater. Express, Vol. 13, Issue 2, pp. 469-483* **2023**, *13* (2), 469–483. https://doi.org/10.1364/OME.478830.

(66) Weng, G. M.; Li, J.; Alhabeb, M.; Karpovich, C.; Wang, H.; Lipton, J.; Maleski, K.; Kong, J.; Shaulsky, E.; Elimelech, M.; Gogotsi, Y.; Taylor, A. D. Layer-by-Layer Assembly of Cross-Functional Semi-Transparent MXene-Carbon Nanotubes Composite Films for Next-Generation Electromagnetic Interference Shielding. *Adv. Funct. Mater.* **2018**, *28* (44), 1803360. https://doi.org/10.1002/ADFM.201803360.
